# Causal relationship between gut microbiota and kidney diseases: a two-sample Mendelian randomization study

**DOI:** 10.3389/fimmu.2023.1277554

**Published:** 2024-01-12

**Authors:** Zhoushan Feng, Yuliang Zhang, Yiyu Lai, Chunhong Jia, Fan Wu, Dunjin Chen

**Affiliations:** ^1^ Department of Obstetrics and Gynecology, Guangdong Provincial Key Laboratory of Major Obstetric Diseases, Guangdong Provincial Clinical Research Center for Obstetrics and Gynecology, Guangdong-Hong Kong-Macao Greater Bay Area Higher Education Joint Laboratory of Maternal-Fetal Medicine, Guangzhou, China; ^2^ Department of Neonatology, Guangzhou Key Laboratory of Neonatal Intestinal Diseases, The Third Affiliated Hospital of Guangzhou Medical University, Guangzhou, China

**Keywords:** Mendelian randomization, gut microbiota, inflammation, kidney diseases, glomerulonephritis, nephrotic syndrome

## Abstract

**Background:**

The interplay between gut microbiome genera and inflammatory kidney-related diseases, such as nephrotic syndrome, glomerulonephritis, tubulo-interstitial nephritis, and chronic kidney disease, has been observed. However, the causal relationships between specific bacterial genera and these renal diseases have not been fully elucidated.

**Objective:**

To investigate the potential causal links between different genera of the gut microbiome and the susceptibility to various renal conditions utilizing two-sample Mendelian randomization (MR) analyses.

**Materials and methods:**

Genome-wide association study (GWAS) summary statistics of gut microbiota and inflammatory kidney-related diseases were obtained from published GWASs. Two-sample MR analyses were conducted using methods including inverse-variance weighted (IVW), MR Egger, and others to identify potential causal links between gut microbial genera and renal conditions. Sensitivity analyses, including Cochran’s Q test and the MR-PRESSO global test, were performed to validate the robustness of the results and detect horizontal pleiotropy. In addition, a reverse MR analysis was conducted to assess reverse causation possibilities.

**Results:**

By synthesizing insights from both primary and sensitivity analyses, this study unveiled critical associations of 12 bacterial genera with nephrotic syndrome, 7 bacterial genera with membranous nephropathy, 3 bacterial genera with glomerulonephritis, 4 bacterial genera with acute tubulo-interstitial nephritis, 6 bacterial genera with chronic tubulo-interstitial nephritis, and 7 bacterial genera with chronic kidney disease. Various genera were pinpointed as having either positive or negative causal relationships with these renal conditions, as evidenced by specific ranges of IVW-OR values (all P< 0.05). The congruence of the sensitivity analyses bolstered the primary findings, displaying no marked heterogeneity or horizontal pleiotropy. Notably, the reverse MR analysis with nephritis as the exposure did not reveal any causal relationships, thereby strengthening the resilience and validity of the primary associations.

**Conclusion:**

This study explored the causal associations between several gut microbial genera and the risk of several inflammatory kidney-related diseases, uncovering several associations between specific gut microbial genera and nephrotic syndrome, membranous nephropathy, glomerulonephritis, tubulo-interstitial nephritis, and chronic kidney disease. These findings enhance our understanding of the complex interplay between the gut microbiome and kidney diseases, and they will be beneficial for early diagnosis and subsequent treatment.

## Introduction

Kidney diseases, characterized by a gradual decline in kidney function and encompassing conditions such as nephrotic syndrome, glomerulonephritis, tubulo-interstitial nephritis, and chronic kidney disease, present substantial health challenges affecting millions globally (1). These ailments are the forefront of morbidity and mortality, with chronic kidney disease alone being an escalating global health concern (2, 3). Notably, these renal conditions frequently progress to end-stage renal diseases, necessitating dialysis or transplantation, and are often intertwined with cardiovascular and immunological complications (4, 5). Despite extensive research, the etiology and progression mechanisms of many kidney diseases remain elusive, and multiple factors, including genetics, environmental triggers, and immune dysregulation, were shown to be implicated in those processes (6, 7).

The gut microbiota, pivotal in regulating host intestinal metabolism, also plays a crucial role in modulating both localized and systemic immune responses of the host (8, 9). Individuals with renal diseases often exhibit a state of subtle inflammation. Dysregulation of the gut microbiota can intensify immune imbalances and foster the production of proinflammatory cytokines, triggering a systemic inflammatory response. This can subsequently expedite the progression of renal diseases and associated cardiovascular complications (10, 11). Recent insights have unveiled a compelling connection between the gut microbiome and kidney health (10). During renal dysfunction, the gut-kidney axis plays a pivotal role, where alterations in gut microbial composition impact kidney function, and reciprocally, renal disturbances can reshape the gut microbial landscape (12, 13). Some studies have indicated that bacteria such as Bifidobacterium exert protective effects against nephropathies (14, 15). However, the literature is riddled with inconsistencies. For instance, while certain investigations attribute kidney-protective roles to bacteria such as Blautia, others posit them as potential renal risk factors (16–18). The majority of preceding studies used a case−control design, making it challenging to ascertain the chronology of exposure and outcomes. Furthermore, observational studies investigating the relationship between the gut microbiota and renal disease are vulnerable to confounders such as age, diet, and lifestyle (19), thus hampering any direct causal conclusions.

In light of these complexities, Mendelian randomization (MR) emerges as an avant-garde method to discern the causal links between the gut microbiome and kidney diseases. By harnessing genetic variants as instrumental variables (IVs), MR can ascertain causal associations between exposures (such as microbial changes) and disease outcomes while remaining unhindered by typical confounding variables (20). The random inheritance of genotypes ensures that any identified associations are less likely to be spurious (21). Leveraging MR, several researchers have unearthed links between gut microbiota and disorders such as metabolic diseases and autoimmune conditions (22, 23). In this study, by harnessing genome-wide association study (GWAS) summary statistics from leading consortiums, we conducted a two-sample MR analysis, aiming to unravel the intricate associations between the gut microbiota and kidney diseases.

## Method

### Study framework

**Figure 1:**
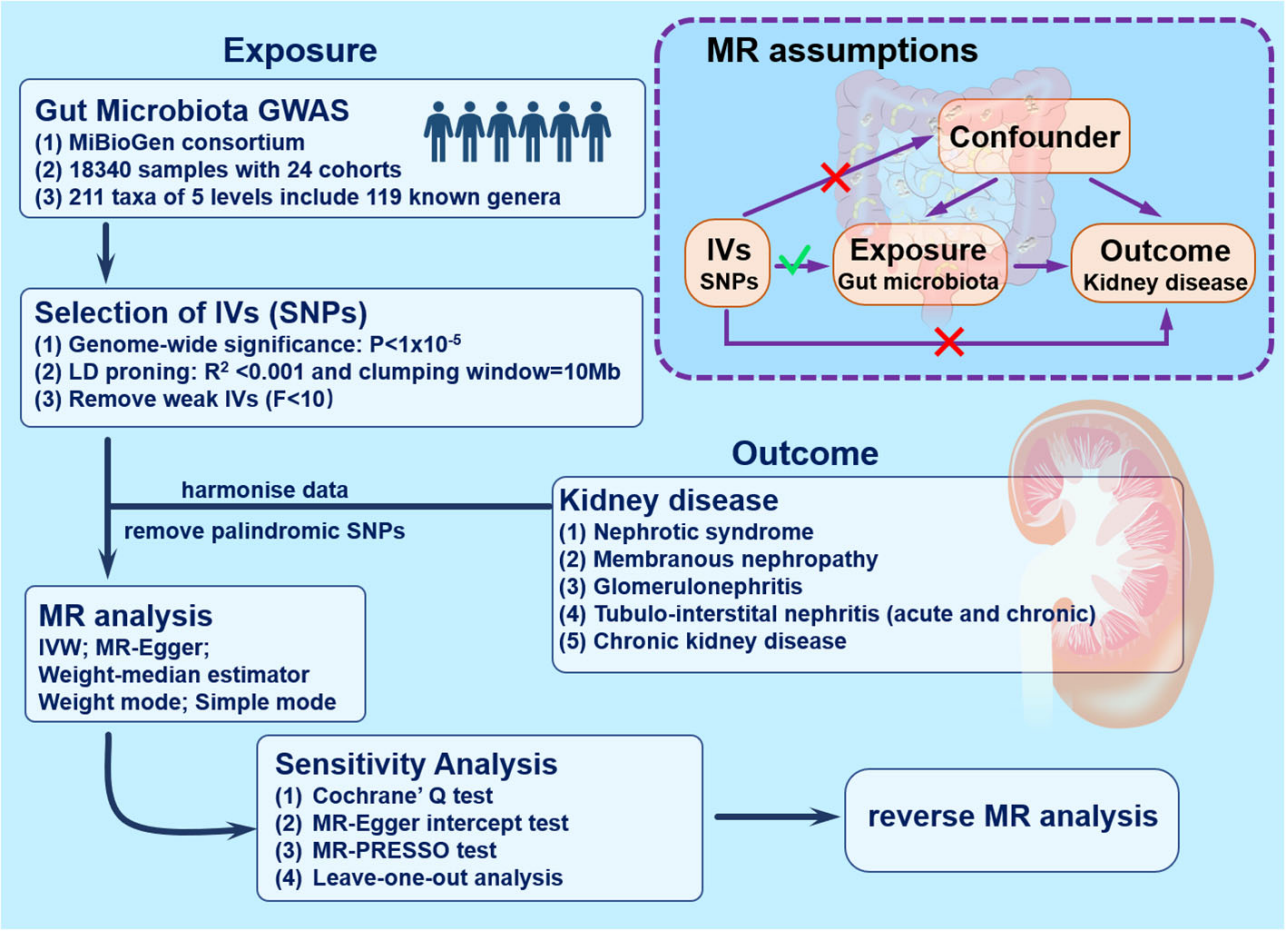
Study design and workflow. GWAS, genome-wide association studies; IVs, instrumental variables; IVW, inverse-variance weighting; MR, mendelian randomization; SNPs, single nucleotide polymorphisms.

The research blueprint is outlined in **Figure 1**. Adopting the two-sample MR technique revealed a connection between genera of the gut microbiome and the susceptibility to renal conditions such as nephrotic syndrome, membranous nephropathy, glomerulonephritis, tubulo-interstitial nephritis (both acute and chronic forms), and chronic kidney disease. In deploying the MR methodology, adherence to three pivotal assumptions was ensured to curtail biases (24). First, there is a notable correlation between the IVs and the gut microbiota. Second, these IVs operate autonomously, implying no ties to external confounders. Last, the IVs, beyond the exposure, should not affect the outcome through any other pathways.

### Data source

Genetic variations of the gut microbiome were derived from the MiBioGen consortium’s extensive genome-wide meta-analysis (25). This research encompassed 18,340 individuals across 24 cohorts, predominantly of European ancestry (n=13,266). Specifically, targeted key regions (V4, V3-V4, and V1-V2) of the 16S rRNA gene provided an in-depth view of the microbiome, with taxa sorted using direct taxonomic binning. Moreover, after adjustments were made for factors such as age, sex, technical covariates, and genetic principal components, Spearman correlation analysis was employed to identify genetic loci impacting the adjusted abundance of bacterial taxa. Additionally, microbiome quantitative trait locus (mbQTL) mapping was utilized to pinpoint host genetic variations influencing the abundance levels of bacterial taxa within the gut microbiome. In this context, ‘genus’ was the smallest and most specific taxonomic level. To optimize precision, analysis was conducted on 131 bacterial taxa solely at the genus level. This group comprised 131 genera, each with an average abundance exceeding 1%, including 12 unidentified genera (25). Finally, a total of 119 genus-level taxa were analyzed (26). More detailed information on the microbiome data can be found on the website (https://mibiogen.gcc.rug.nl/) and elsewhere (25). The GWAS data for membranous nephropathy was sourced from datasets imported from the European bioinformatics institute (EBI) database, whose datasets satisfy the minimum requirements for complete GWAS summary data. Summary statistics for GWAS of nephrotic syndrome, glomerulonephritis, tubulointerstitial nephritis (both acute and chronic), and chronic kidney disease were derived from the FinnGen biobank. For ease of analysis and data extraction, we obtained and analyzed the outcome data directly through the OPEN GWAS website (https://gwas.mrcieu.ac.uk/). Detailed information about the collected data, such as the ID numbers, number of patients, control participants, single nucleotide polymorphisms (SNPs), and sample sizes, could be found in **Supplementary Table 1**.

### Selection criteria for instrumental variables

In the process of selecting IVs, we employed a comprehensive set of criteria to ensure the robustness and reliability of our analysis: (1) Genome-wide significance threshold: SNPs associated with each genus reaching a genome-wide significance threshold (P < 1.0×10^-5^) were identified as potential IVs. This stringent threshold ensures the selection of SNPs with a strong association with the respective genera (27). (2) Strength assessment using F-statistic: To address weak instrument bias, we employed the F-statistic (F = β² exposure/SE² exposure) to evaluate the strength of each IV (8, 21, 28). SNPs with an F-statistic below 10 were excluded to minimize the impact of weak instruments (29). It is essential to note that, even in double-sample MR, where instrument bias is toward null, we still consider F-statistic filtering necessary (30). (3) Independence and linkage disequilibrium mitigation (LD): To ensure the independence of selected variables and mitigate LD effects, we implemented two criteria. Firstly, an R² threshold of 0.001 was set for SNPs, ensuring a high degree of independence among chosen variables. Additionally, a genetic distance of 10 Mb was enforced, further reducing potential LD effects. (4) Minor allele frequency (MAF) exclusion: SNPs with MAF ≤ 0.01 were systematically excluded. This criterion helps focus on common genetic variants, ensuring the stability and generalizability of the instrumental variables selected for the analysis. (5) Handling palindromic SNPs: For palindromic SNPs, where the strand directionality is ambiguous, allele frequency data were utilized to infer the forward strand allele. This approach ensures consistency in the interpretation of allele information for these specific SNPs.

### Mendelian randomization analysis

To investigate the causal relationship between exposure and outcome, various methods, including inverse-variance weighted (IVW), MR Egger, weighted median, weighted mode, and simple mode, were employed. The IVW method, a traditional approach and the primary method used in this study, combines the Wald ratio estimates of each IV in a meta-analysis, which is equivalent to a weighted linear regression of the relationship between the IVs and the outcome with the intercepts of the IVs constrained to zero. IVW is advantageous, as it provides unbiased estimates when no horizontal pleiotropy exists. Conversely, the MR Egger approach relies on the instrument strength independent of direct effect (InSIDE) assumption. This assumption entails that the IVs used should be strongly associated with the exposure but should not have a direct effect on the outcome, independent of the exposure. This is critical in ensuring that the relationships captured reflect the dose-response between the IVs and the outcome, while also accounting for potential pleiotropic effects (31). The weighted median method reduces the occurrence of type-1 errors, allowing for some invalid genetic variants. The weighted mode and simple mode methods remain reliable when the majority of IVs with similar causal estimates are valid, even if some do not meet the requirements for causal inference. In the event of inconsistent results across these methods, IVW results are prioritized as the primary outcome. For consistency, summary statistics were harmonized to ensure that each IV was correlated with the same effect allele. Ambiguous SNPs, such as A/T and C/G alleles that could potentially lead to confusion in their linkage properties, were removed, and the data were then aligned accordingly. In terms of sensitivity analyses, Cochran’s Q test was employed to assess heterogeneity among the IVs, while leave-one-out sensitivity analysis gauged the influence of outliers and result stability. The presence of horizontal pleiotropy could challenge the second MR hypothesis; thus, various methods were applied to detect potential horizontal pleiotropy. Specifically, the MR−Egger intercept test and the MR-PRESSO global test evaluated the presence of horizontal pleiotropy with p < 0.05 considered to indicate statistical significance (32, 33). MR-PRESSO adjusts for horizontal pleiotropy by identifying and removing outliers (34), setting the distribution count to 1000 in the MR-PRESSO analysis (35). In the reverse MR analysis, nephritis was considered the exposure and the established causal bacterial genera was considered the outcome. SNPs associated with nephritis served as IVs. All statistical analyses were performed using R software version 4.2.3, utilizing the ‘MR-PRESSO’ and ‘TwoSampleMR’ packages.

## Result

Adhering to the selection standards for IVs, a total of 1232 SNPs were utilized as IVs for 119 different bacterial genera. These SNPs were selected based on parameters including genome-wide significance level (p < 1×10^-5^), LD test results, harmonization processes, and F-statistic validations. All IVs exhibited F-statistics between 14.59 and 35.26, exceeding the threshold of 10, thereby establishing a robust association with their respective bacterial taxonomic units. Thus, the study effectively bypassed the pitfall of weak IV bias. Specifically, key associations were identified (IVW-P < 0.05): 121 human genomic SNPs showing statistical associations with 12 bacterial genera were linked to nephrotic syndrome; similarly, 58 SNPs associated with 7 genera to membranous nephropathy; 34 SNPs with 3 genera to glomerulonephritis; 34 SNPs with 4 genera to acute tubulo-interstitial nephritis; 70 SNPs with 6 genera to chronic tubulo-interstitial nephritis; and 79 SNPs with 7 genera to chronic kidney disease. It is important to clarify that these identified SNPs are part of the human genome and their association with specific bacterial genera is inferred based on statistical correlations, not direct genetic contributions from the bacteria (**Supplementary Table 2**).

### Nephrotic syndrome

**Figure 2:**
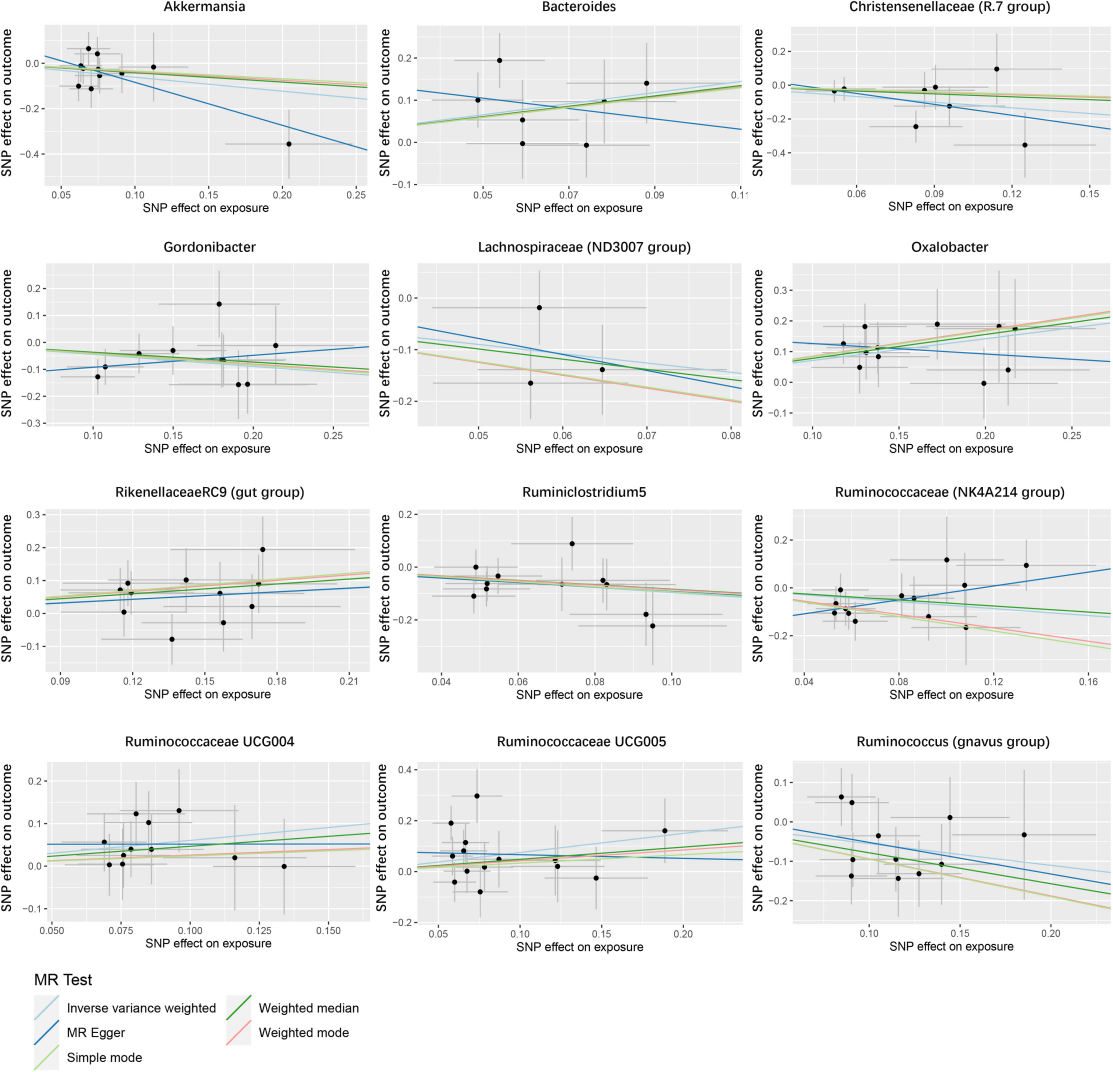
Scatter plot of the causal association between gut microbiome and nephrotic syndrome. SNPs = single nucleotide polymorphisms.

**Figure 3:**
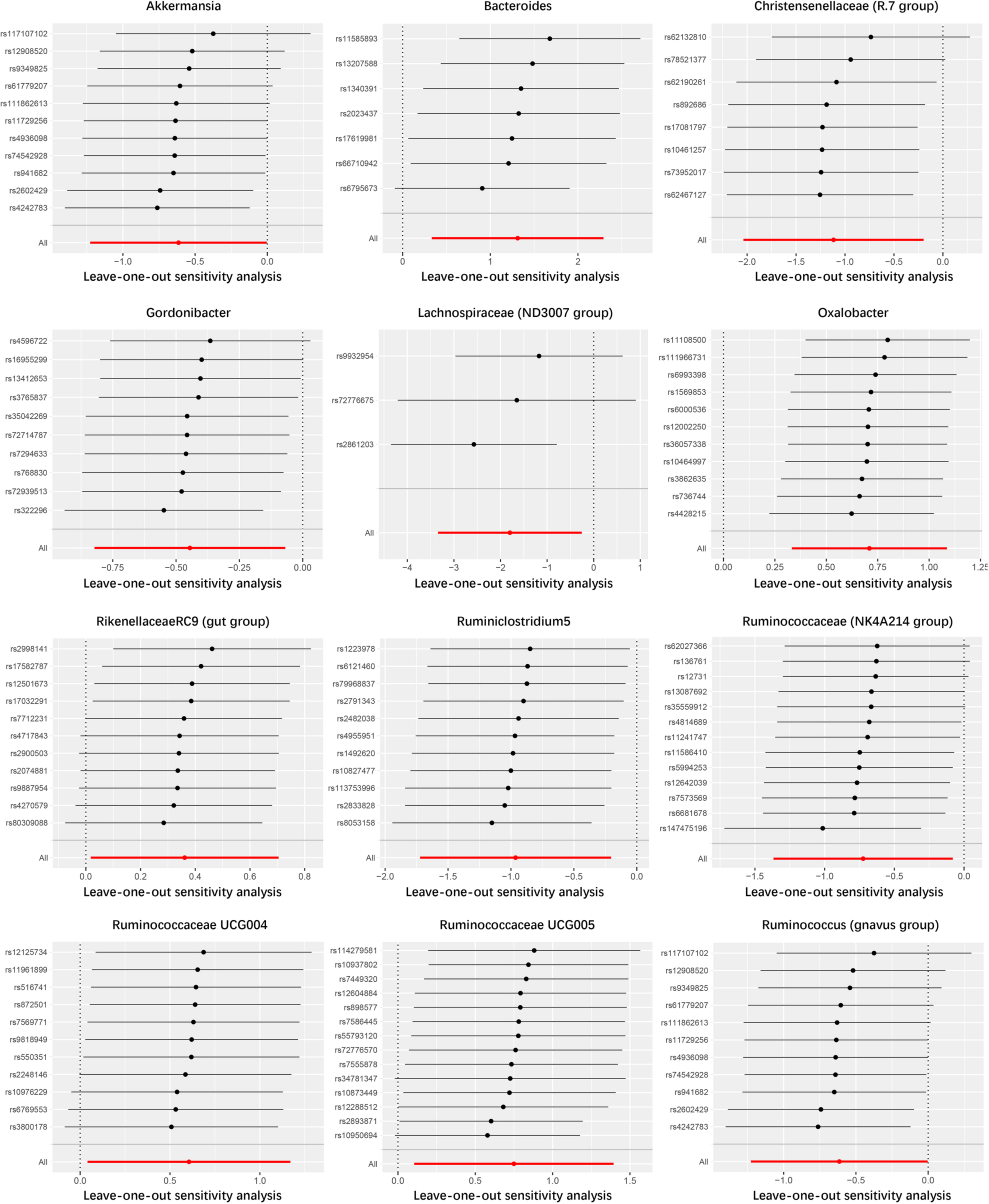
Leave-one-out analysis of the causal association between gut microbiome and nephrotic syndrome.

In the study of the relationship between gut microbiota and nephrotic syndrome, 12 genera of bacteria were found to have a causal association with the disease (IVW-P< 0.05). Specifically, Bacteroides, Oxalobacter, Rikenellaceae RC9 (gut group), Ruminococcaceae UCG004, and Ruminococcaceae UCG005 were associated with an increased risk of nephrotic syndrome. Their IVW-ORs (95% Cis) were 3.71 (1.39, 9.90), 2.03 (1.39, 2.96), 1.44 (1.02, 2.02), 1.83 (1.04, 3.22), and 2.12 (1.11, 4.03), respectively. Conversely, Ruminococcus (gnavus group), Akkermansia, Christensenellaceae (R-7 group), Gordonibacter, Lachnospiraceae (ND3007 group), and Ruminococcaceae (NK4A214 group) were associated with a decreased risk of nephrotic syndrome, with IVW-ORs (95% CI) of 0.58 (0.36, 0.91), 0.54 (0.29, 1.00), 0.33 (0.13, 0.82), 0.64 (0.44, 0.93), 0.17 (0.04, 0.77), 0.38 (0.18, 0.82), and 0.48 (0.25, 0.92), respectively (**Table 1**). In most MR analyses, the trends in MR Egger, weighted median, weighted mode, and simple mode aligned with IVW. However, for Bacteroides, Gordonibacter, Oxalobacter, and Ruminococcaceae (NK4A214 group), MR Egger showed a contrasting trend (**Figure 2**). Leave-one-out plots showed potential outliers in the IVs for specific bacterial groups (**Figure 3**). Nevertheless, neither significant heterogeneity in Cochran’s Q test of the IVW results (P > 0.05) nor evident directional horizontal pleiotropy in MR−Egger regression (P > 0.05) or MR-PRESSO analyses were detected (global test P > 0.05) (**Supplementary Tables 3-5**).

**Table 1:** MR estimates for the association between gut microbiota and nephrotic syndrome.

Bacterial genus(exposure)	No. of SNP	MR method	OR	95% CI	P-value
**Akkermansia**	11	MR Egger	0.15	(0.02, 1.16)	0.10
		Weighted median	0.66	(0.28, 1.57)	0.35
		IVW	0.54	(0.29, 1.00)	0.049
		Simple mode	0.71	(0.17, 2.90)	0.64
		Weighted mode	0.69	(0.15, 3.15)	0.64
**Bacteroides**	7	MR Egger	0.29	(0.00, 36.39)	0.64
		Weighted median	3.40	(0.94, 12.27)	0.06
		IVW	3.71	(1.39, 9.90)	0.01
		Simple mode	3.25	(0.43, 24.46)	0.30
		Weighted mode	3.30	(0.47, 23.02)	0.27
**Christensenellaceae (R.7 group)**	8	MR Egger	0.12	(0.00, 2.98)	0.24
		Weighted median	0.56	(0.16, 1.96)	0.37
		IVW	0.33	(0.13, 0.82)	0.02
		Simple mode	0.65	(0.10, 4.02)	0.66
		Weighted mode	0.62	(0.10, 3.71)	0.62
**Gordonibacter**	10	MR Egger	1.56	(0.34, 7.08)	0.58
		Weighted median	0.69	(0.42, 1.14)	0.15
		IVW	0.64	(0.44, 0.93)	0.02
		Simple mode	0.66	(0.32, 1.37)	0.29
		Weighted mode	0.67	(0.31, 1.44)	0.33
**Lachnospiraceae (ND3007 group)**	3	MR Egger	–	–	–
		Weighted median	0.14	(0.02, 0.98)	0.05
		IVW	0.17	(0.04, 0.77)	0.02
		Simple mode	0.09	(0.01, 1.03)	0.19
		Weighted mode	0.08	(0.006, 1.06)	0.20
**Oxalobacter**	11	MR Egger	0.71	(0.12, 4.18)	0.71
		Weighted median	2.18	(1.30, 3.65)	0.003
		IVW	2.03	(1.39, 2.96)	0.00023
		Simple mode	2.30	(0.97, 5.44)	0.09
		Weighted mode	2.34	(0.96, 5.71)	0.09
**Rikenellaceae RC9 (gut group)**	11	MR Egger	1.46	(0.17, 12.41)	0.74
		Weighted median	1.65	(1.05, 2.59)	0.03
		IVW	1.44	(1.02, 2.02)	0.04
		Simple mode	1.79	(0.84, 3.81)	0.16
		Weighted mode	1.75	(0.81, 3.77)	0.19
**Ruminiclostridium5**	11	MR Egger	0.43	(0.02, 10.25)	0.61
		Weighted median	0.43	(0.15, 1.23)	0.12
		IVW	0.38	(0.18, 0.82)	0.01
		Simple mode	0.39	(0.08, 2.03)	0.29
		Weighted mode	0.43	(0.11, 1.71)	0.26
**Ruminococcaceae (NK4A214 group)**	13	MR Egger	4.28	(0.51, 35.73)	0.21
		Weighted median	0.53	(0.21, 1.32)	0.17
		IVW	0.48	(0.25, 0.92)	0.03
		Simple mode	0.22	(0.05, 1.03)	0.08
		Weighted mode	0.25	(0.05, 1.26)	0.12
**Ruminococcaceae UCG004**	11	MR Egger	1.00	(0.04, 23.24)	1.00
		Weighted median	1.60	(0.77, 3.32)	0.21
		IVW	1.83	(1.04, 3.22)	0.04
		Simple mode	1.27	(0.40, 4.06)	0.69
		Weighted mode	1.30	(0.46, 3.68)	0.64
**Ruminococcaceae UCG005**	14	MR Egger	0.87	(0.15, 4.93)	0.87
		Weighted median	1.62	(0.73, 3.58)	0.24
		IVW	2.12	(1.11, 4.03)	0.02
		Simple mode	1.39	(0.39, 4.95)	0.62
		Weighted mode	1.53	(0.52, 4.54)	0.46
**Ruminococcus (gnavus group)**	11	MR Egger	0.45	(0.05, 4.07)	0.49
		Weighted median	0.46	(0.24, 0.87)	0.02
		IVW	0.58	(0.36, 0.91)	0.02
		Simple mode	0.39	(0.13, 1.16)	0.12
		Weighted mode	0.39	(0.14, 1.10)	0.10

CI, confidence interval; IVW, inverse-variance weighting; MR, mendelian randomization; OR, odds ratio; SNPs, single nucleotide polymorphisms.

### Membranous nephropathy

The results revealed positive associations of the bacterial genera Butyricicoccus, Butyrivibrio, Catenibacterium, Ruminiclostridium5, and Ruminococcaceae UCG003 with membranous nephropathy, suggesting a causal link to an increased risk of this disease (IVW-OR (95% CI): 2.16 (1.01, 4.62), 1.25 (1.00, 1.57), 1.49 (1.04, 2.13), 1.74 (1.05, 2.86), 1.78 (1.14, 2.76)). Conversely, the negative association of Oscillibacter and Ruminococcaceae UCG013 with membranous nephropathy indicated that they are causally associated with a reduced risk of this condition (IVW-OR (95% CI): 0.53 (0.29, 0.96), 0.57 (0.33, 1.00)) (**Supplementary Table 6**). Potential outliers in the IVs of Butyricicoccus, Butyrivibrio, Oscillibacter, and Ruminococcaceae UCG003 were visible in scatter and leave-one-out plots (**Supplementary Figures 1**, **2**). Heterogeneity analysis using Cochran’s Q test revealed variability only in the Oscillibacter genus (P < 0.05), with other genera showing no heterogeneity (**Supplementary Table 3**). Additionally, no significant horizontal pleiotropy was found in MR−Egger regression (P >0.05) (**Supplementary Table 4**). After MR-PRESSO analysis, only Oscillibacter showed horizontal pleiotropy, which violated assumption 3. Excluding the SNP rs9393920, which was previously shown to be associated with mineral metabolism and potential kidney damage (36), reduced this pleiotropy significantly (**Supplementary Table 5**).

### Glomerulonephritis

Two bacterial genera, Coprococcus3 and Oxalobacter, were found to have detrimental effects, with IVW-OR (95% CI) values of 1.36 (1.03, 1.81) and 1.20 (1.07, 1.37), respectively. In contrast, Erysipelotrichaceae UCG003 appeared to have a protective effect (0.74 (0.61, 0.90)) (**Supplementary Table 7**). Cochran’s Q test results showed no significant heterogeneity (P>0.05), and MR−Egger regression intercept analysis showed no significant directional horizontal pleiotropy (**Supplementary Tables 3, 4**). Even though potential outliers were identified in scatter and leave-one-out plots (**Supplementary Figures 3**, **4**), MR-PRESSO analysis showed no significant outliers (P>0.05) (**Supplementary Table 5**). Therefore, there is insufficient evidence to indicate horizontal pleiotropy in the associations between these bacteria and glomerulonephritis.

### Tubulo-interstitial nephritis

Investigations into tubulointerstitial nephritis separated analyses into acute and chronic disease courses.

In acute tubulo-interstitial nephritis, MR analysis revealed that Marvinbryantia and Odoribacter increased disease risk with IVW-ORs (95% CI) of 1.13 (1.13, 1.52) and 1.23 (1.03, 1.48), respectively, while Actinomyces and Gordonibacter decreased disease risk, represented by IVW-ORs of 0.84 (0.74, 0.94) and 0.90 (0.82, 0.97), respectively (**Supplementary Table 8**, **Supplementary Figure 5**).

For chronic tubulo-interstitial nephritis, IVW MR analysis demonstrated a causal link between increased disease risk and the presence of Coprococcus3, Intestinimonas, and Victivallis, showing ORs (95% CI) of 2.90 (1.35, 6.22), 1.58 (1.01, 2.47), and 1.44 (1.04, 1.99), respectively. In contrast, Dorea, Erysipelotrichaceae UCG003 and Eubacterium (brachy group) were causally linked to a reduction in disease risk, with ORs (95% CIs) of 0.46 (0.22, 0.93), 0.57 (0.35, 0.94) and 0.70 (0.50, 1.00), respectively (**Supplementary Table 9**, **Supplementary Figure 6**).

Sensitivity analyses, including Cochran’s Q test for IVW, MR−Egger regression intercept analysis and MR-PRESSO analysis, revealed no significant heterogeneity or horizontal pleiotropy among the IVs (P> 0.05) (**Supplementary Tables 3-5**, **Supplementary Figures 7**, **8**).

### Chronic kidney disease

Butyrivibrio, Coprococcus3, Oxalobacter, Prevotella7, and Ruminococcus2 were found to be causally associated with an increased risk of chronic kidney disease (IVW-OR (1.11 (1.00, 1.24), 1.36 (1.00, 1.85), 1.19 (1.04, 1.36), 1.16 (1.02, 1.33) and 1.24 (1.01, 1.53), respectively). Conversely, Erysipelotrichaceae UCG003 and Lachnospira (0.73 (0.59, 0.90) and 0.64 (0.42, 0.96)) were associated with a reduced risk of chronic kidney disease (**Supplementary Table 10**, **Supplementary Figure 9**). The analysis showed no significant heterogeneity according to Cochran’s Q test and no directional horizontal pleiotropy as per the MR-PRESSO test (**Supplementary Tables 3-5**, **Supplementary Figure 10**).

### Reverse MR analysis

Finally, reverse MR analysis was performed on the gut microbiota genera identified to have an impact on kidney disease, with nephritis as the exposure and the identified pathogenic bacterial genera as the outcome. However, no causal relationships were found in the analysis (IVW-P >0.05) (**Supplementary Table 11**).

## Discussion

This comprehensive investigation has revealed an array of intricate relationships between specific bacterial genera and various kidney diseases by employing stringent selection criteria for IVs. The study found 121 SNPs linked to 12 bacterial genera associated with nephrotic syndrome; 58 SNPs linked to 7 genera with membranous nephropathy; 34 SNPs linked to 3 genera with glomerulonephritis; 34 SNPs linked to 4 genera with acute tubulo-interstitial nephritis; 70 SNPs linked to 6 genera with chronic tubulo-interstitial nephritis; and 79 SNPs linked to 7 genera with chronic kidney disease. These findings indicate statistical links between human genomic SNPs and specific bacterial genera, related to different kidney disorders. Various analytical methodologies were employed to ensure the robustness of the associations, including different MR methods, Cochran’s Q test, MR−Egger regression intercept analysis, and MR-PRESSO analysis. Notably, the study bypassed the pitfall of weak IV bias and further identified possible horizontal pleiotropy and outliers, adding layers of reliability and depth to the research. These insights not only enhance our understanding of the connection between gut microbiota and renal health but also herald new pathways for therapeutic interventions and personalized medicine in nephrology.

Several studies have unveiled the complex relationship between gut microbiota and various renal disorders (17, 37–39). In our comprehensive investigation, specific bacterial genera linked to various kidney diseases were identified. Notable examples such as Ruminococcaceae (including subtypes UCG003-5 and Ruminococcus2) and other genera such as Coprococcus3 and Oxalobacter were consistently associated with adverse effects on renal health. Other research has linked Ruminococcaceae and Ruminococcus to an increased risk of nephrotic syndrome and elevated blood urea nitrogen levels (40). Additionally, Ruminococcus was found in higher abundance in patients with membranous nephropathy with nephrotic syndrome (41). A reduced abundance of Coprococcus3 was observed to exert protective effects on chronic kidney disease (42), and Bacteroides abundance noticeably decreased following treatment for nephrotic syndrome. Interestingly, while other renal disease studies have portrayed Butyrivibrio as having a protective role (43, 44), our research uniquely identified a link between Butyrivibrio and an elevated risk of membranous nephropathy and chronic kidney disease through MR analysis. This discovery merits further in-depth investigation in future studies. These studies emphasize the pathogenic roles of these gut microbiotas in the onset of renal diseases.

Beneficial gut bacteria play diverse roles that could potentially offer therapeutic targets in renal disorders. In our research, specific bacterial taxa demonstrated protective roles across a spectrum of kidney diseases. Erysipelotrichaceae UCG003 was found to confer protective effects against glomerulonephritis, chronic tubulo-interstitial nephritis, and chronic kidney disease, while Gordonibacter showed protection against nephrotic syndrome and acute tubulo-interstitial nephritis. Similarly, other bacterial strains, such as Ruminococcaceae UCG013 and Lachnospira, played a protective role in different renal conditions. Notably, Ruminococcaceae UCG013 showed increased abundance following treatment for nephrotic syndrome (45), while Erysipelotrichaceae demonstrated protective effects in IgA nephropathy (46) and was associated with a delay in renal failure, fibrosis, and inflammation (47). Genera such as Lachnospira and Ruminococcus (gnavus group) have been identified as significant markers that differentiate healthy individuals from those with chronic kidney disease (48). These insights are in line with a growing body of research underscoring the multifaceted roles of beneficial gut bacteria in health. This nuanced comprehension of the gut-kidney axis not only highlights its significance as a pioneering field in renal medicine but also signals the necessity for further investigation to uncover novel therapeutic approaches. Furthermore, the modulation of the gut microbiome through probiotic or prebiotic interventions emerges as an exciting prospect for treating renal diseases (49).

Gut microbiota may contribute to kidney disease through diverse pathways. On one hand, altered intestinal permeability enables bacterial translocation, instigating systemic inflammation and oxidative stress, which can potentially compromise renal function (50). Maintaining intestinal barrier integrity is thus crucial to prevent harmful bacterial transference (51). For example, Akkermansia is considered beneficial for gut health, particularly in maintaining the mucosal layer, thereby reducing the release of gut-derived toxins that could damage the kidneys (52, 53). On the other hand, the gut microbiota may influence kidney health through other systemic diseases. It can regulate the endocrine system, affecting kidney diseases through common endocrine disorders such as diabetes or obesity, which are high-risk factors for renal health (54). In our study, Dorea bacteria were shown to reduce the risk of chronic tubulo-interstitial nephritis, potentially linked to their direct impact on glucose metabolism (55). Furthermore, the gut microbiome can impact renal health through systems like the immune system. In our study, Lachnospira demonstrated a protective causal effect against chronic kidney disease. Similarly, another MR analysis found that Lachnospira could lower the risk of systemic lupus erythematosus, a significant risk factor for kidney damage (56, 57). There are also other reports highlighting the role of the gut microbiota in modulating the immune response to suppress inflammation, thereby lessening the impact on chronic kidney disease (12, 58). Recent analyses have also uncovered a close relationship between the gut microbiome and cardiovascular diseases, such as pre-eclampsia and hypertension, which can notably impact kidney health (26, 59). The gut-kidney axis might also regulate hypertension and cardiovascular issues through its influence on salt sensitivity and vascular function. These diseases similarly have a marked impact on renal health (60).

Additionally, bacteria or their metabolic products may regulate or disrupt renal metabolism. Our study confirmed that Bacteroides could increase the risk of nephrotic syndrome and elevate the release of indoxyl sulfate, leading to kidney damage (61, 62), and are highly correlated with the stages of chronic kidney disease (63). Likewise, Ruminiclostridium 5 in our study is linked to a reduced risk of nephrotic syndrome. Although current systematic research on Ruminiclostridium 5 is limited, some studies suggest its enrichment is associated with lowered blood calcium levels, thereby reducing the risk of kidney stones, indicating its potential impact on electrolyte balance (64). Furthermore, the gut microbiome has been shown to impact renal metabolites, such as uric acid and urea, among others (65, 66). Indeed, the interactions between the gut microbiome and kidney diseases through their metabolic products or other systems mechanisms warrant further exploration through mediation MR studies to better understand their causal relationships.

Our research has uncovered that bacteria not extensively reported in the literature also have a causal impact on renal health, such as Intestinimonas and Rikenellaceae RC9 (gut group). Overall, these bacteria may affect kidney health through mechanisms such as protecting the gut barrier, immune modulation (67), endocrine regulation (68), or secretion of metabolites (69). Beyond gut bacteria, it is also important to consider the overall abundance of bacteria and their impact on other gut microbes, like fungi and viruses (70). There are reports indicating that gut viruses can directly infect kidney cells or induce renal damage through causing hypertension (71, 72) and fungi can harm the kidneys by triggering immune inflammation (73, 74). These factors deserve attention in future research and clinical work.

This study has several distinctive strengths. MR analysis was conducted to ascertain the causal relationship between the gut microbiota and kidney diseases, effectively mitigating the interference of confounding variables and potential reverse causation. Genetic variants related to gut microbiota were gathered from the most extensive available GWAS meta-analysis, reinforcing the robustness of the IVs used in the MR analysis. Techniques such as MR-PRESSO and MR−Egger regression intercept term tests were utilized to detect and eliminate horizontal pleiotropy. Furthermore, inverse MR was employed to determine the presence of any reverse causal relationship. By adopting a two-sample MR design and utilizing nonoverlapping exposure and outcome summary-level data, bias was minimized in the study, and a rigorous framework was established for exploring the complex connections between gut microbiota and renal disorders (75).

However, several limitations in this study warrant attention and could affect the interpretation of the results. Utilizing summary statistics rather than individual-level data limited the ability to perform nuanced analyses, such as differentiating between stages of kidney diseases or investigating nonlinear relationships. The analysis was further constrained by the genus-level resolution of the exposure dataset, which precluded exploration of causal associations between specific gut microbiota species and kidney diseases. Moreover, the relatively small sample size and the inclusion of SNPs that did not meet traditional GWAS significance thresholds (P < 5×10^−8^) might have introduced bias into the results of this study. Additionally, the predominance of European participants in the GWAS meta-analysis may have introduced population stratification interference, thereby limiting the applicability of the findings to other ethnic groups. This study identified a direct causal relationship between the gut microbiome and kidney health but did not delve into whether this relationship is mediated through the immune system or metabolic pathways. To provide more comprehensive insights into the causal relationship between gut microbiota and kidney diseases, future MR studies should address these limitations, possibly by incorporating more diverse populations and refining the taxonomic levels in the analysis, and by employing mediation MR methods.

## Conclusion

This MR study reveals the complex relationship between gut microbiota and different kidney disorders. Various bacterial genera were identified as having causal associations with an increased or decreased risk of nephrotic syndrome, membranous nephropathy, glomerulonephritis, tubulo-interstitial nephritis, and chronic kidney disease. Intricate links were consistently found across these disorders, with minor heterogeneity and horizontal pleiotropy observed in some cases. These findings offer a glimpse into the potential for early disease diagnosis and therapeutic targets in kidney diseases, thus highlighting the need for further refined research to fully exploit these associations for clinical benefit.

## Data availability statement

The original contributions presented in the study are included in the article/**Supplementary Material**. Further inquiries can be directed to the corresponding authors.

## Author contributions

ZF: Conceptualization, Data curation, Formal analysis, Investigation, Methodology, Software, Writing – original draft. YZ: Conceptualization, Data curation, Formal analysis, Writing – original draft. YL: Conceptualization, Data curation, Investigation, Methodology, Writing – original draft. CJ: Data curation, Methodology, Software, Writing – original draft. FW: Funding acquisition, Resources, Writing – review & editing. DC: Funding acquisition, Project administration, Resources, Supervision, Validation, Writing – review & editing.

## Supplementary material

Supplementary Figure 1 Scatter plot of the causal association between gut microbiome and membranous nephropathy. SNPs = single nucleotide polymorphisms.

Supplementary Figure 2 Leave-one-out analysis of the causal association between gut microbiome and membranous nephropathy.

Supplementary Figure 3 Scatter plot of the causal association between gut microbiome and glomerulonephritis. SNPs = single nucleotide polymorphisms.

Supplementary Figure 4 Leave-one-out analysis of the causal association between gut microbiome and glomerulonephritis.

Supplementary Figure 5 Scatter plot of the causal association between gut microbiome and acute tubulo-interstitial nephritis. SNPs = single nucleotide polymorphisms.

Supplementary Figure 6 Scatter plot of the causal association between gut microbiome and chronic tubulo-interstitial nephritis. SNPs = single nucleotide polymorphisms.

Supplementary Figure 7 Leave-one-out analysis of the causal association between gut microbiome and acute tubulo-interstitial nephritis.

Supplementary Figure 8 Leave-one-out analysis of the causal association between gut microbiome and chronic tubulo-interstitial nephritis.

Supplementary Figure 9 Scatter plot of the causal association between gut microbiome and chronic kidney disease. SNPs = single nucleotide polymorphisms.

Supplementary Figure 10 Leave-one-out analysis of the causal association between gut microbiome and chronic kidney disease.
