# Supplementary figures and images for: Causal relationship between gut microbiota and kidney diseases: a two-sample Mendelian randomization study
Source: Front Immunol. 2024 Jan 12;14:1277554. doi: 10.3389/fimmu.2023.1277554 (PMC10811222; doi:10.3389/fimmu.2023.1277554)

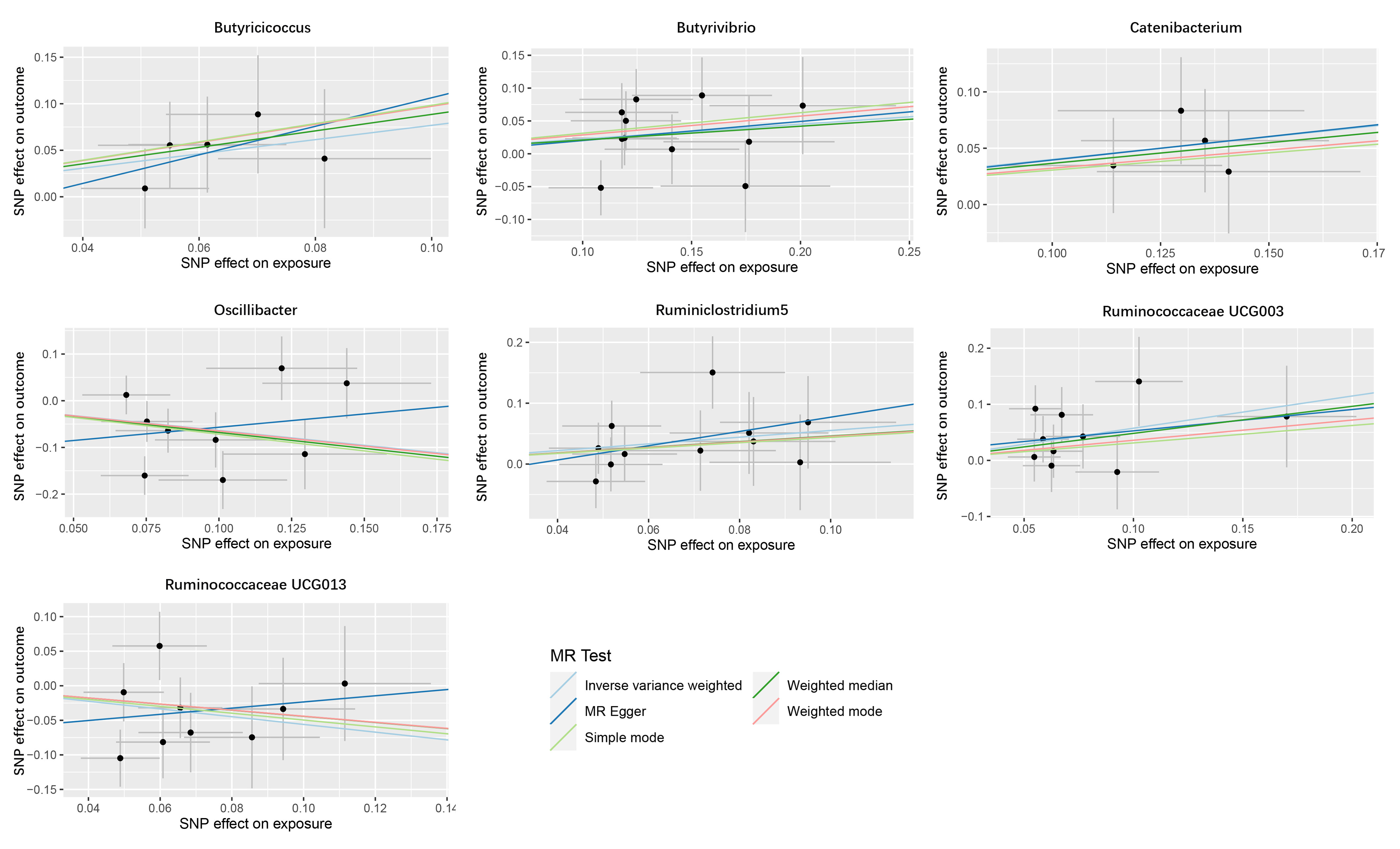

Supplement: Supplementary Figure 1 — Scatter plot of the causal association between gut microbiome and membranous nephropathy. SNPs = single nucleotide polymorphisms. [file Image_1.jpeg]

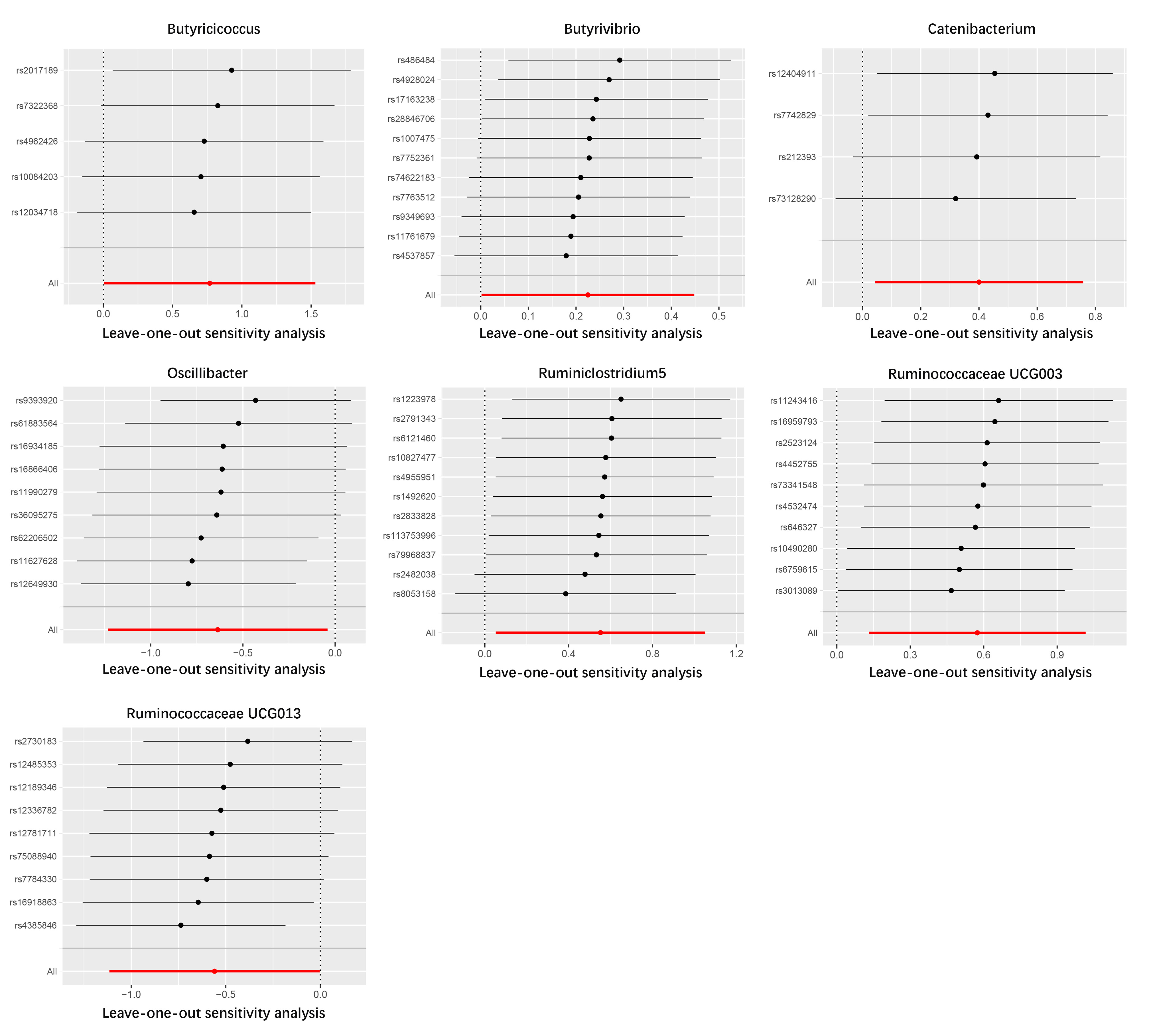

Supplement: Supplementary Figure 2 — Leave-one-out analysis of the causal association between gut microbiome and membranous nephropathy. [file Image_2.jpeg]

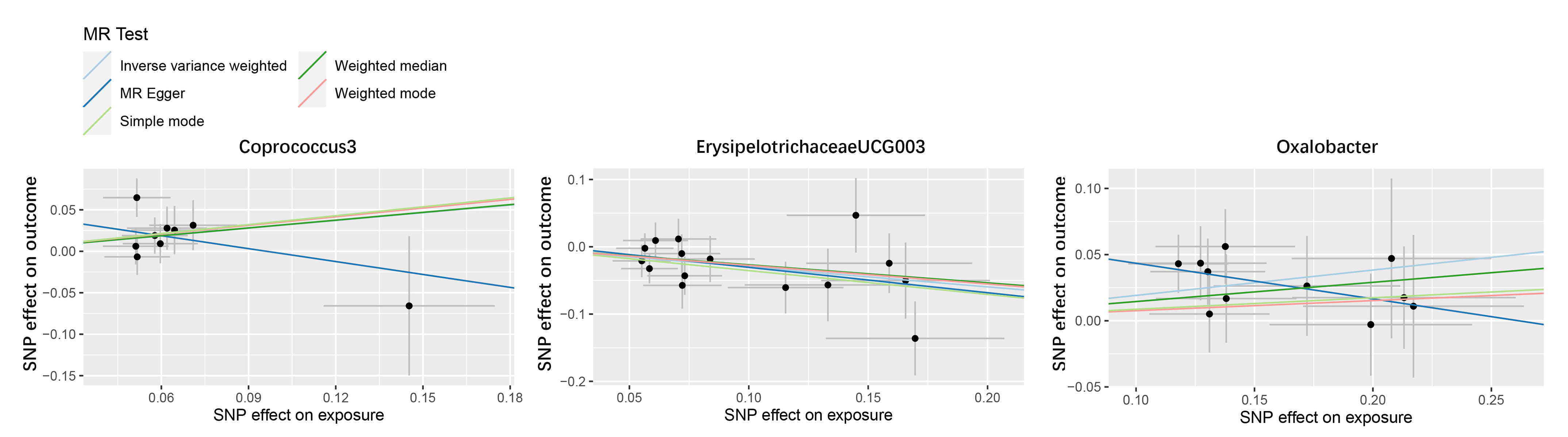

Supplement: Supplementary Figure 3 — Scatter plot of the causal association between gut microbiome and glomerulonephritis. SNPs = single nucleotide polymorphisms. [file Image_3.jpeg]

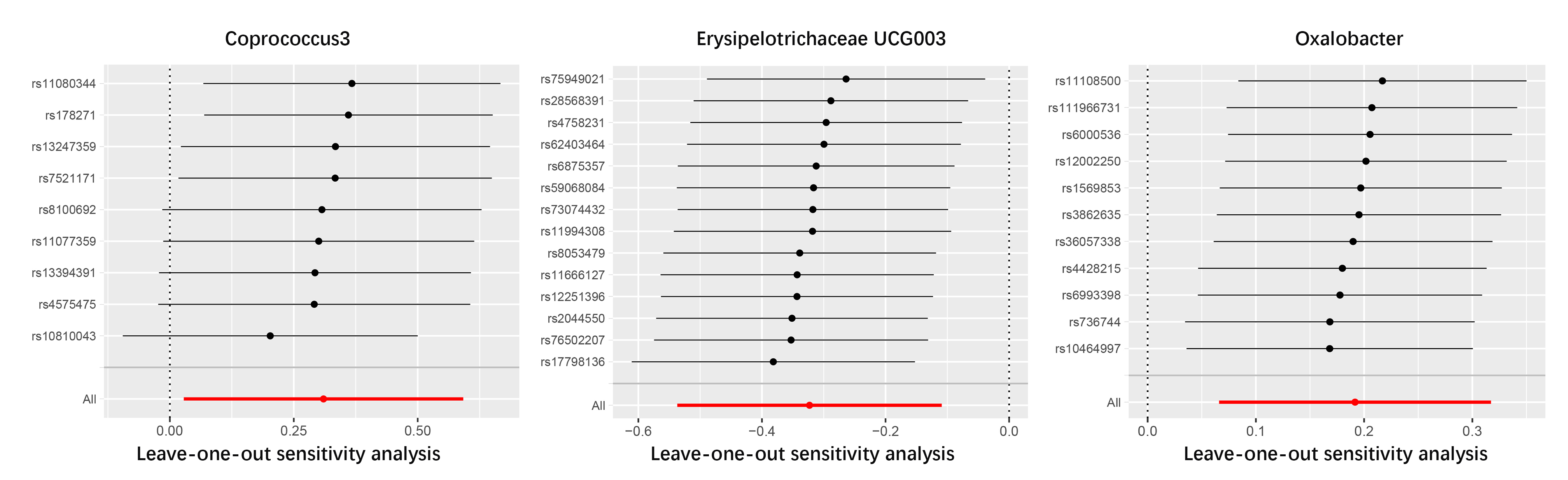

Supplement: Supplementary Figure 4 — Leave-one-out analysis of the causal association between gut microbiome and glomerulonephritis. [file Image_4.jpeg]

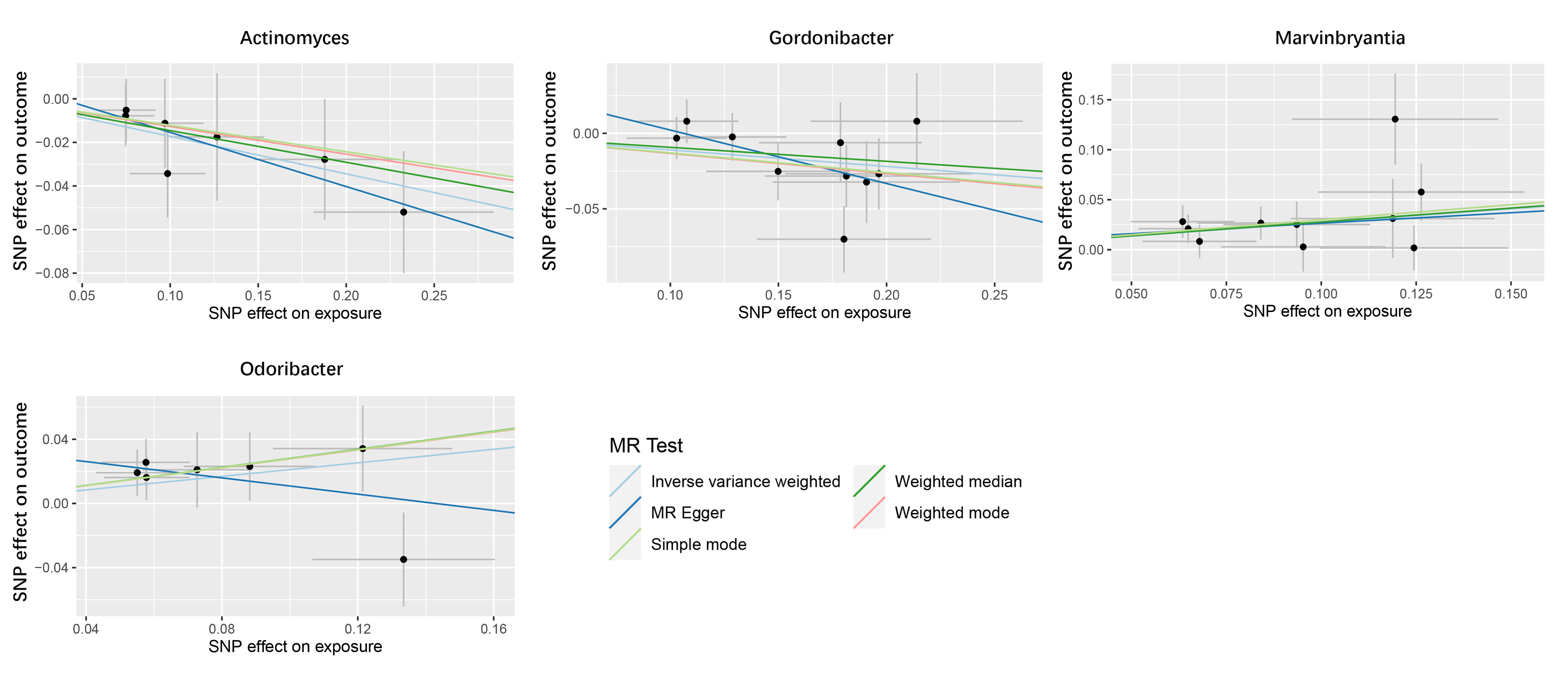

Supplement: Supplementary Figure 5 — Scatter plot of the causal association between gut microbiome and acute tubulo-interstitial nephritis. SNPs = single nucleotide polymorphisms. [file Image_5.jpeg]

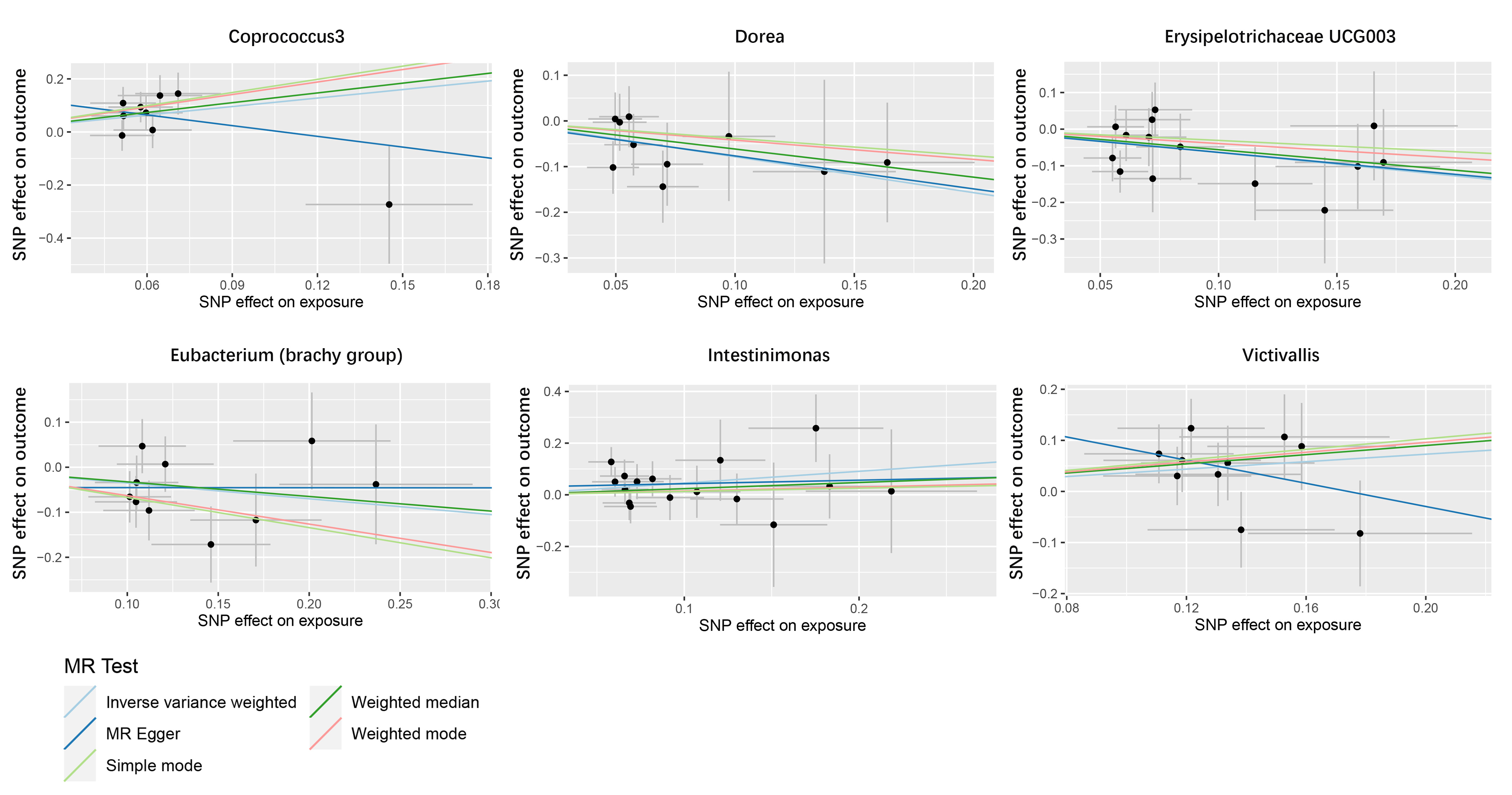

Supplement: Supplementary Figure 6 — Scatter plot of the causal association between gut microbiome and chronic tubulo-interstitial nephritis. SNPs = single nucleotide polymorphisms. [file Image_6.jpeg]

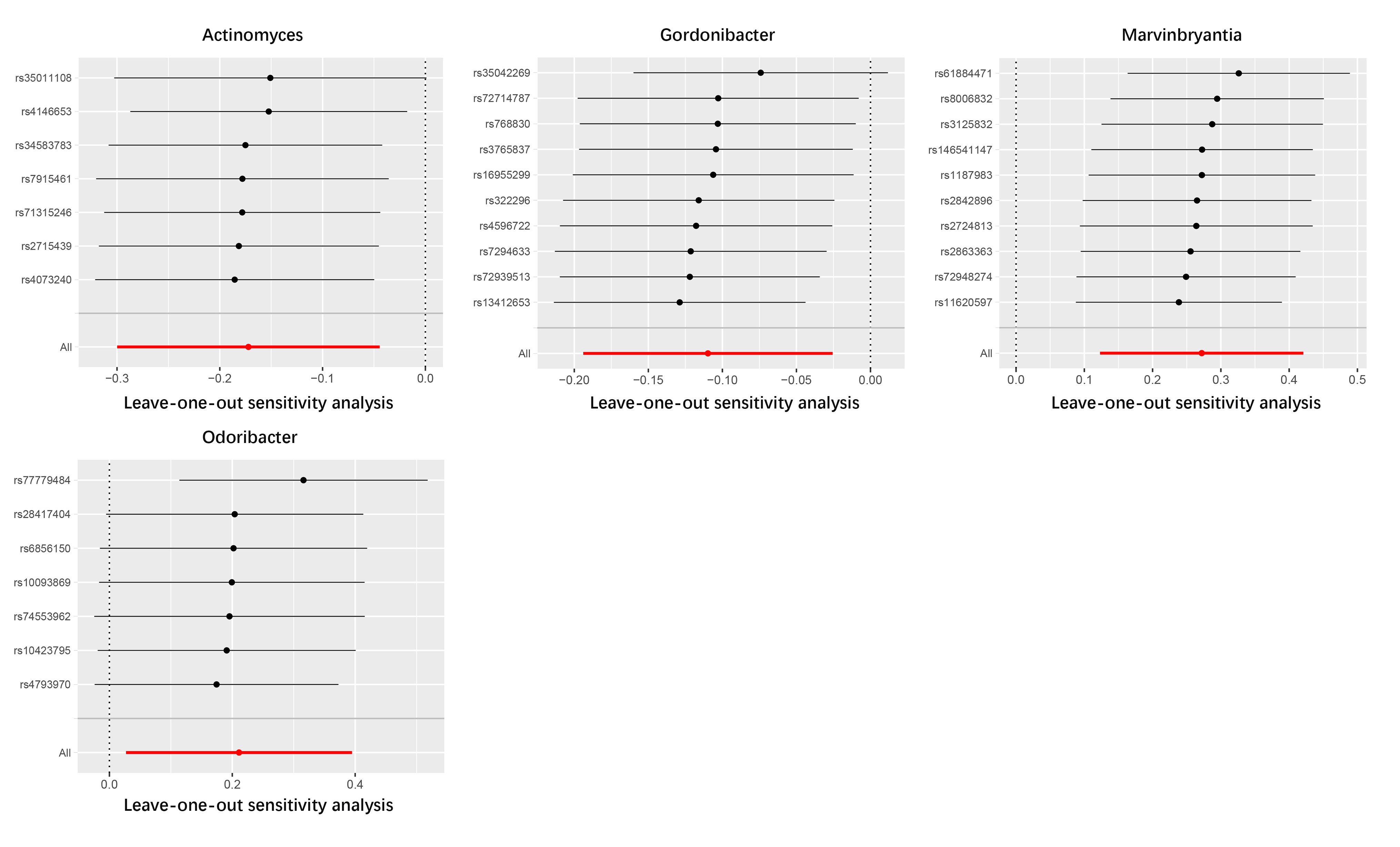

Supplement: Supplementary Figure 7 — Leave-one-out analysis of the causal association between gut microbiome and acute tubulo-interstitial nephritis. [file Image_7.jpeg]

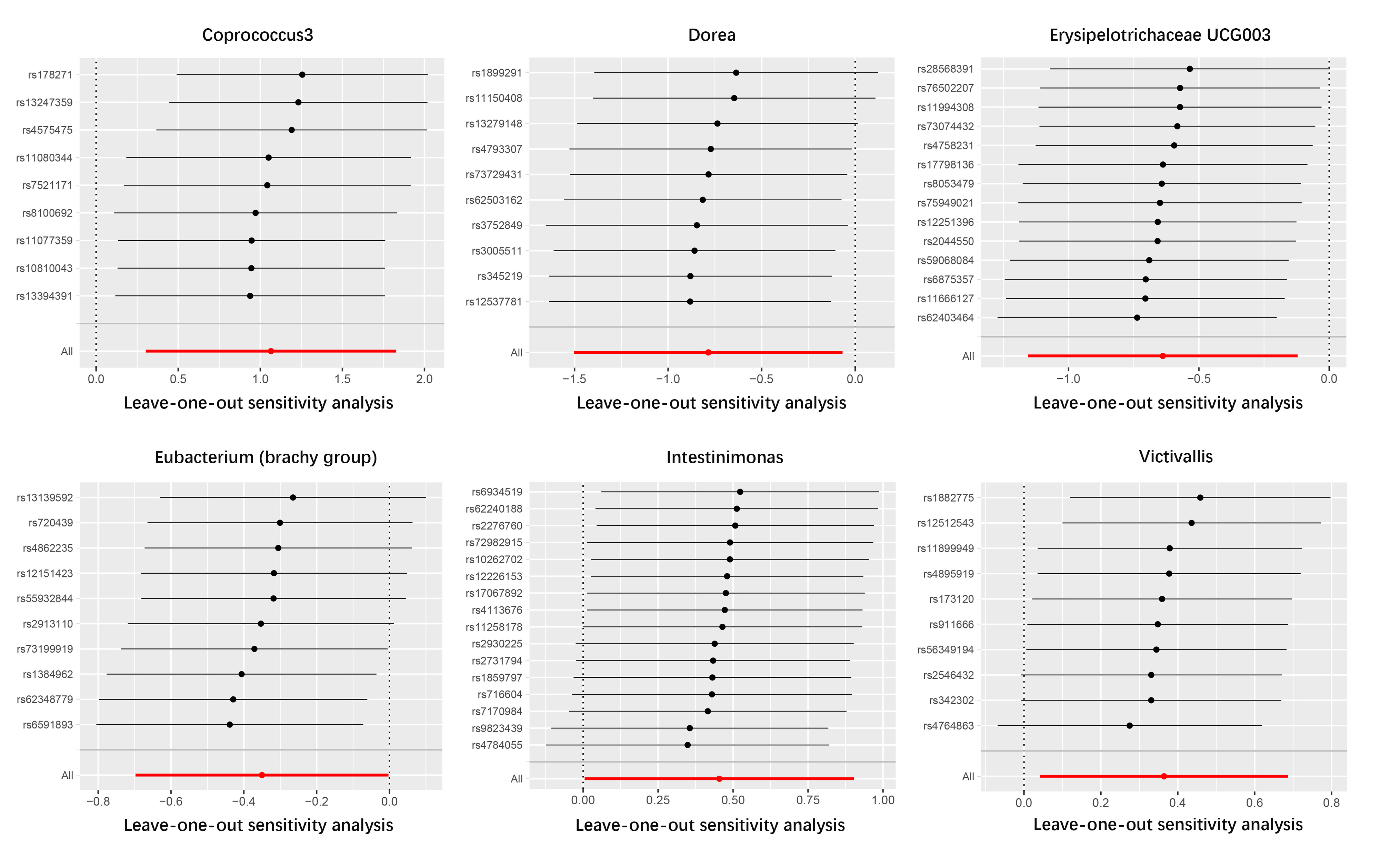

Supplement: Supplementary Figure 8 — Leave-one-out analysis of the causal association between gut microbiome and chronic tubulo-interstitial nephritis. [file Image_8.jpeg]

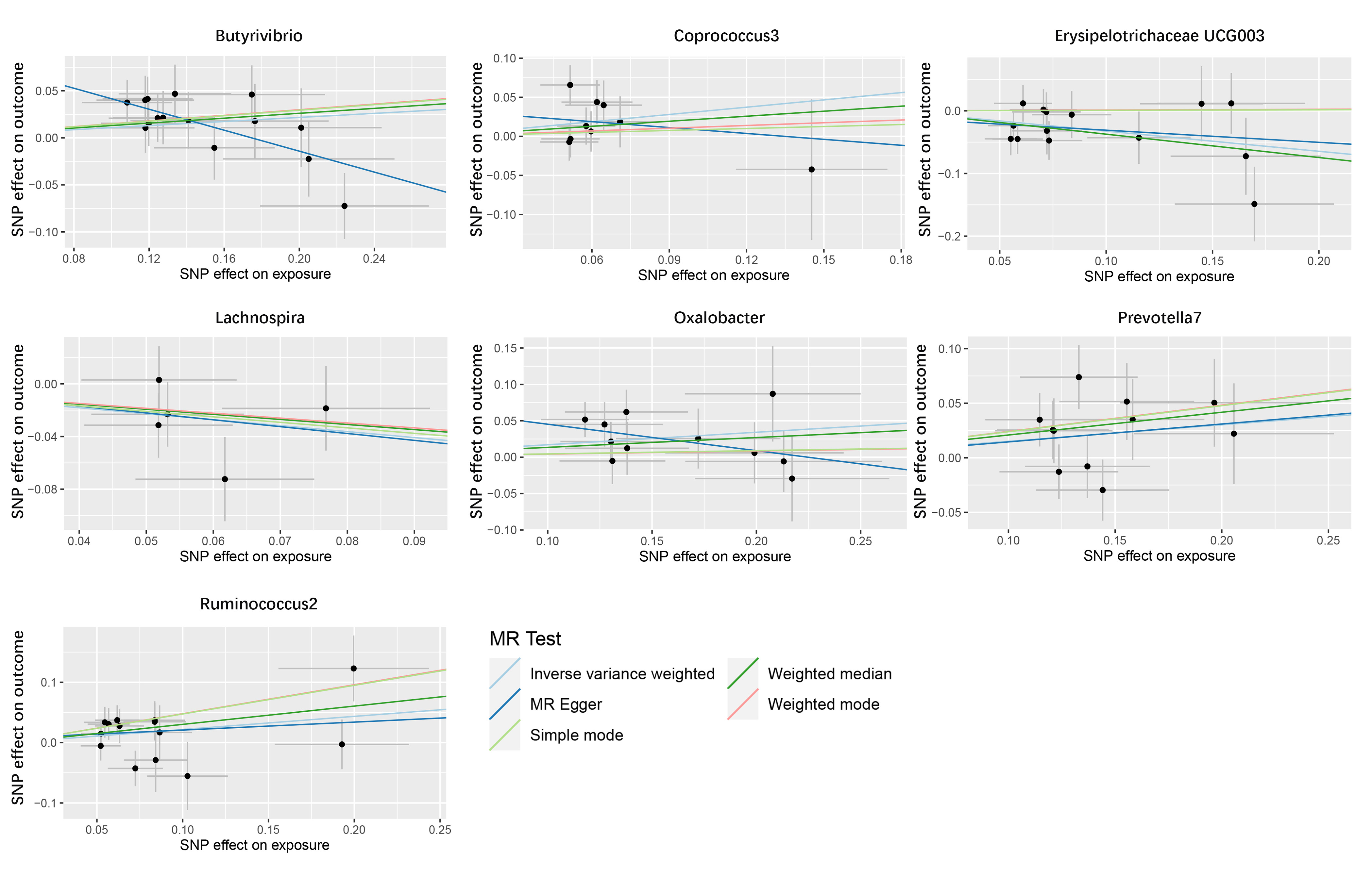

Supplement: Supplementary Figure 9 — Scatter plot of the causal association between gut microbiome and chronic kidney disease. SNPs = single nucleotide polymorphisms. [file Image_9.jpeg]

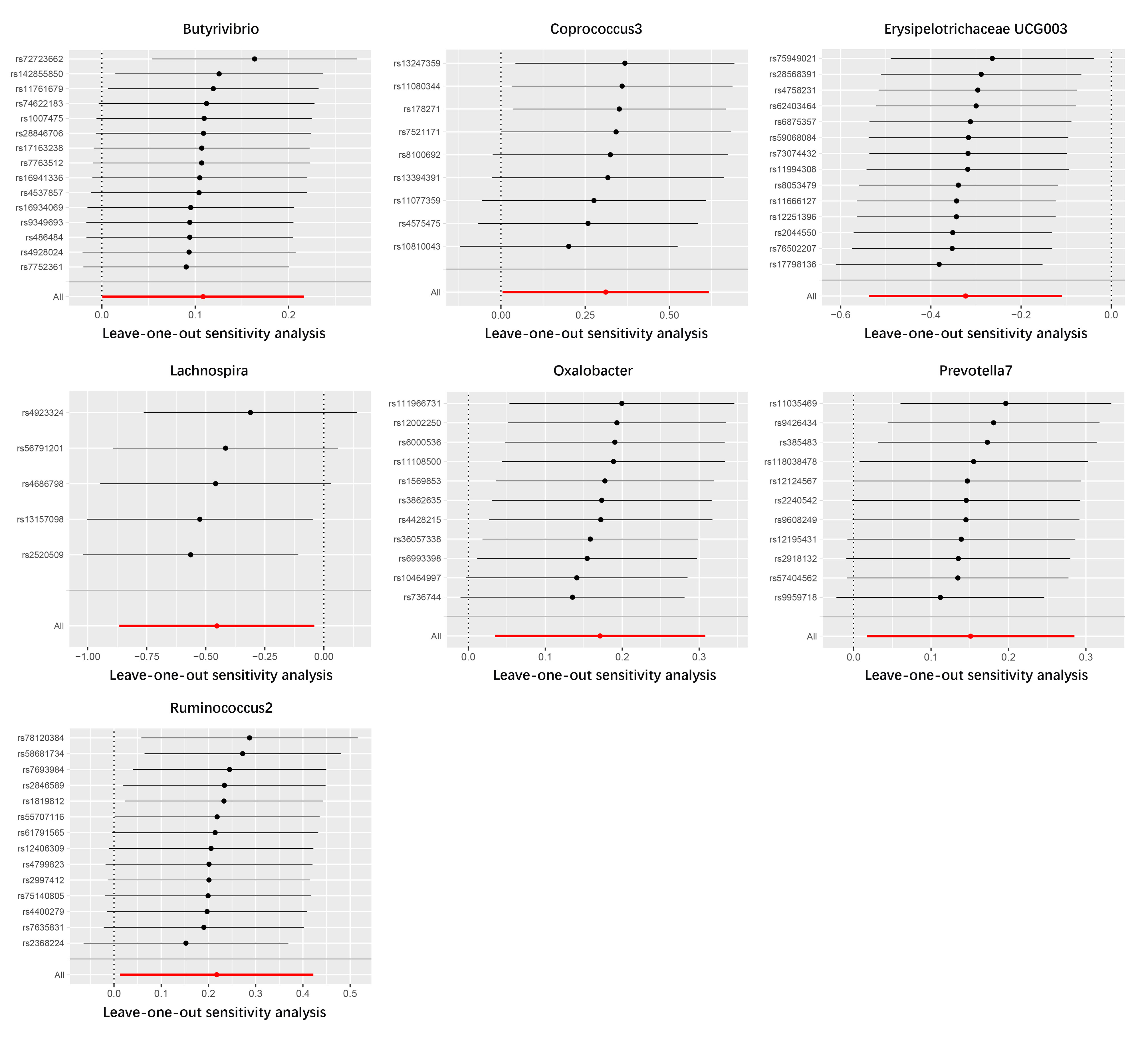

Supplement: Supplementary Figure 10 — Leave-one-out analysis of the causal association between gut microbiome and chronic kidney disease. [file Image_10.jpeg]
