# Supplementary material for: Causal relationship between gut microbiota and kidney diseases: a two-sample Mendelian randomization study
Source: Front Immunol. 2024 Jan 12;14:1277554. doi: 10.3389/fimmu.2023.1277554 (PMC10811222; doi:10.3389/fimmu.2023.1277554)
Supplement: Supplementary file 11 [file Table_1.docx]

**Supplementary materials**

**Page 2. Supplemental Table 1.** Data source and GWAS sample size for different renal disorders

**Page 3. Supplemental Table 2.** Utilizing instrumental variables in MR analysis to explore the link between gut microbiota and kidney disease.

**Page 17. Supplemental Table 3.** Heterogeneity in instrumental variables for gut microbiota

**Page 18. Supplemental Table 4.** Assessment of horizontal pleiotropy in the connection between gut microbiota and kidney disease using MR Egger regression

**Page 19. Supplemental Table 5.** Analyzing the link between gut microbiota and kidney disease with MR-PRESSO

**Page 21. Supplemental Table 6.** MR estimates for the association between gut microbiota and membranous nephropathy

**Page 22. Supplemental Table 7.** MR estimates for the association between gut microbiota and glomerulonephritis

**Page 23. Supplemental Table 8.** MR estimates for the association between gut microbiota and acute tubulo-interstitial nephritis

**Page 24. Supplemental Table 9.** MR estimates for the association between gut microbiota and chronic tubulo-interstitial nephritis

**Page 25. Supplemental Table 10.** MR estimates for the association between gut microbiota and chronic kidney disease

**Supplemental Table 1. Data Source and GWAS Sample Size for Different Renal Disorders**

| **Disease** | **ID number**  (https://gwas.mrcieu.ac.uk/) | **Case (n)** | **Control (n)** | **Simple size (n)** | **Number of SNPs (n)** |
| --- | --- | --- | --- | --- | --- |
| **Nephrotic syndrome** | finn-b-N14_NEPHROTICSYND | 480 | 214,619 | 215,099 | 16,380,437 |
| **Glomerulonephritis** | finn-b-GLOMER_NEPHRITIS | 4,613 | 214,179 | 218,792 | 16,380,466 |
| **Acute tubulo-interstitial nephritis** | finn-b-N14_PYELONEPHR | 11,216 | 201,028 | 212,244 | 16,380,447 |
| **Chronic tubulo-interstitial nephritis** | finn-b-N14_CHRONTUBULOINTNEPHRITIS | 620 | 201,028 | 20,748 | 16,380,412 |
| **Chronic kidney disease** | finn-b-N14_CHRONKIDNEYDIS | 3,902 | 212,841 | 216,743 | 16,380,459 |
| **Membranous nephropathy** | [ebi-a-GCST010005](https://gwas.mrcieu.ac.uk/datasets/ebi-a-GCST010005/) | 2,150 | 5,829 | 7,979 | 5,327,688 |

GWAS = genome-wide association studies; SNPs = single nucleotide polymorphisms.

**Supplemental Table 2. Utilizing instrumental variables in MR analysis to explore the link between gut microbiota and kidney disease.**

| **Kidney disease** | **Bacterial (exposure)** | **SNP** | **Effect allele** | **Other allele** | **F** | **Exposure** | | | **Outcome** | | |
| --- | --- | --- | --- | --- | --- | --- | --- | --- | --- | --- | --- |
|  |  |  |  |  |  | **Beta** | **SE** | **P-value** | **Beta** | **SE** | **P-value** |
| **Nephrotic syndrome** | |  |  |  |  |  |  |  |  |  |  |
| 1 | **Akkermansia** | rs111862613 | T | C | 19.30 | 0.099 | 0.021 | 4.50E-06 | 0.029 | 0.028 | 0.29 |
| 2 |  | rs117107102 | A | G | 21.07 | 0.188 | 0.040 | 5.92E-06 | 0.097 | 0.037 | 0.01 |
| 3 |  | rs11729256 | T | C | 20.31 | 0.075 | 0.016 | 6.27E-06 | -0.035 | 0.019 | 0.07 |
| 4 |  | rs12908520 | G | A | 29.66 | 0.233 | 0.051 | 6.34E-06 | -0.047 | 0.038 | 0.22 |
| 5 |  | rs2602429 | C | T | 24.59 | 0.075 | 0.017 | 7.94E-06 | 0.013 | 0.020 | 0.52 |
| 6 |  | rs4242783 | G | A | 21.68 | -0.097 | 0.022 | 9.83E-06 | 0.018 | 0.028 | 0.52 |
| 7 |  | rs4936098 | A | G | 19.77 | -0.083 | 0.017 | 9.77E-07 | 0.027 | 0.020 | 0.18 |
| 8 |  | rs61779207 | G | A | 21.86 | -0.119 | 0.025 | 2.05E-06 | 0.038 | 0.032 | 0.23 |
| 9 |  | rs74542928 | T | C | 19.56 | -0.073 | 0.016 | 2.54E-06 | 0.004 | 0.019 | 0.82 |
| 10 |  | rs9349825 | A | G | 20.93 | 0.143 | 0.031 | 4.81E-06 | 0.023 | 0.035 | 0.51 |
| 11 |  | rs941682 | G | A | 20.93 | -0.095 | 0.021 | 5.23E-06 | 0.043 | 0.027 | 0.12 |
| 1 | **Bacteroides** | rs11585893 | A | G | 25.18 | -0.07 | 0.01 | 5.24E-07 | 0.01 | 0.08 | 0.93 |
| 2 |  | rs13207588 | A | G | 20.36 | -0.06 | 0.01 | 6.40E-06 | 0.00 | 0.08 | 0.97 |
| 3 |  | rs1340391 | T | C | 20.04 | -0.06 | 0.01 | 7.58E-06 | -0.05 | 0.10 | 0.58 |
| 4 |  | rs17619981 | T | G | 22.19 | 0.09 | 0.02 | 2.46E-06 | 0.14 | 0.10 | 0.14 |
| 5 |  | rs2023437 | T | C | 21.78 | -0.08 | 0.02 | 3.06E-06 | -0.10 | 0.10 | 0.33 |
| 6 |  | rs66710942 | C | T | 20.64 | 0.05 | 0.01 | 5.53E-06 | 0.10 | 0.07 | 0.12 |
| 7 |  | rs6795673 | C | T | 26.18 | 0.05 | 0.01 | 3.11E-07 | 0.19 | 0.07 | 0.00 |
| 1 | **Christensenellaceae (R.7group)** | rs10461257 | A | G | 20.45 | -0.06 | 0.01 | 6.13E-06 | 0.02 | 0.07 | 0.75 |
| 2 |  | rs17081797 | A | G | 19.60 | -0.09 | 0.02 | 9.53E-06 | 0.01 | 0.13 | 0.93 |
| 3 |  | rs62132810 | A | G | 21.29 | -0.08 | 0.02 | 3.94E-06 | 0.25 | 0.09 | 0.01 |
| 4 |  | rs62190261 | A | C | 19.92 | 0.10 | 0.02 | 8.07E-06 | -0.12 | 0.12 | 0.29 |
| 5 |  | rs62467127 | C | T | 20.51 | 0.11 | 0.03 | 5.94E-06 | 0.10 | 0.21 | 0.65 |
| 6 |  | rs73952017 | C | T | 19.68 | -0.09 | 0.02 | 9.16E-06 | 0.03 | 0.11 | 0.78 |
| 7 |  | rs78521377 | C | T | 20.69 | 0.12 | 0.03 | 5.40E-06 | -0.35 | 0.19 | 0.07 |
| 8 |  | rs892686 | A | G | 21.31 | 0.05 | 0.01 | 3.90E-06 | -0.03 | 0.07 | 0.60 |
| 1 | **Gordonibacter** | rs13412653 | A | C | 20.23 | 0.11 | 0.02 | 6.87E-06 | -0.09 | 0.07 | 0.18 |
| 2 |  | rs16955299 | G | A | 20.53 | -0.20 | 0.04 | 5.88E-06 | 0.16 | 0.11 | 0.16 |
| 3 |  | rs322296 | G | A | 22.44 | 0.18 | 0.04 | 2.17E-06 | 0.14 | 0.12 | 0.25 |
| 4 |  | rs35042269 | C | A | 19.99 | -0.18 | 0.04 | 7.80E-06 | 0.06 | 0.10 | 0.53 |
| 5 |  | rs3765837 | T | G | 19.35 | -0.19 | 0.04 | 1.09E-05 | 0.16 | 0.13 | 0.22 |
| 6 |  | rs4596722 | A | G | 19.75 | 0.10 | 0.02 | 8.83E-06 | -0.13 | 0.07 | 0.05 |
| 7 |  | rs72714787 | C | A | 23.14 | 0.18 | 0.04 | 1.51E-06 | -0.07 | 0.10 | 0.49 |
| 8 |  | rs72939513 | A | G | 19.03 | -0.21 | 0.05 | 1.29E-05 | 0.01 | 0.15 | 0.94 |
| 9 |  | rs7294633 | C | T | 26.50 | 0.13 | 0.02 | 2.63E-07 | -0.04 | 0.07 | 0.57 |
| 10 |  | rs768830 | G | A | 20.21 | 0.15 | 0.03 | 6.93E-06 | -0.03 | 0.09 | 0.74 |
| 1 | **Lachnospiraceae (ND3007 group)** | rs2861203 | G | A | 20.22 | 0.06 | 0.01 | 6.91E-06 | -0.02 | 0.07 | 0.79 |
| 2 |  | rs72776675 | T | C | 19.12 | -0.06 | 0.01 | 1.23E-05 | 0.14 | 0.09 | 0.11 |
| 3 |  | rs9932954 | A | G | 23.47 | -0.06 | 0.01 | 1.27E-06 | 0.16 | 0.07 | 0.02 |
| 1 | **Oxalobacter** | rs10464997 | G | A | 21.81 | 0.14 | 0.03 | 3.00E-06 | 0.11 | 0.08 | 0.18 |
| 2 |  | rs11108500 | A | G | 21.71 | -0.20 | 0.04 | 3.17E-06 | 0.00 | 0.12 | 0.98 |
| 3 |  | rs111966731 | T | C | 20.42 | 0.21 | 0.05 | 6.22E-06 | 0.04 | 0.12 | 0.73 |
| 4 |  | rs12002250 | A | C | 21.68 | 0.22 | 0.05 | 3.22E-06 | 0.17 | 0.16 | 0.28 |
| 5 |  | rs1569853 | T | C | 21.62 | -0.14 | 0.03 | 3.33E-06 | -0.08 | 0.10 | 0.40 |
| 6 |  | rs36057338 | G | T | 24.32 | 0.21 | 0.04 | 8.15E-07 | 0.18 | 0.18 | 0.32 |
| 7 |  | rs3862635 | C | T | 19.09 | -0.17 | 0.04 | 1.25E-05 | -0.19 | 0.11 | 0.10 |
| 8 |  | rs4428215 | G | A | 28.93 | 0.13 | 0.02 | 7.50E-08 | 0.18 | 0.07 | 0.02 |
| 9 |  | rs6000536 | C | T | 26.64 | -0.13 | 0.03 | 2.45E-07 | -0.10 | 0.09 | 0.27 |
| 10 |  | rs6993398 | G | A | 20.81 | 0.13 | 0.03 | 5.06E-06 | 0.05 | 0.08 | 0.56 |
| 11 |  | rs736744 | C | T | 31.14 | 0.12 | 0.02 | 2.41E-08 | 0.13 | 0.07 | 0.06 |
| 1 | **RikenellaceaeRC9 (gut group)** | rs12501673 | A | G | 19.70 | 0.12 | 0.03 | 9.07E-06 | 0.00 | 0.07 | 0.95 |
| 2 |  | rs17032291 | T | C | 21.21 | -0.17 | 0.04 | 4.13E-06 | -0.02 | 0.10 | 0.83 |
| 3 |  | rs17582787 | A | G | 21.53 | -0.16 | 0.03 | 3.48E-06 | 0.03 | 0.09 | 0.75 |
| 4 |  | rs2074881 | T | C | 19.28 | -0.14 | 0.03 | 1.13E-05 | -0.10 | 0.10 | 0.29 |
| 5 |  | rs2900503 | G | T | 27.83 | -0.17 | 0.03 | 1.33E-07 | -0.09 | 0.09 | 0.31 |
| 6 |  | rs2998141 | T | C | 21.64 | -0.14 | 0.03 | 3.28E-06 | 0.08 | 0.08 | 0.31 |
| 7 |  | rs4270579 | G | A | 18.96 | -0.12 | 0.03 | 1.33E-05 | -0.09 | 0.07 | 0.19 |
| 8 |  | rs4717843 | G | T | 20.99 | -0.12 | 0.03 | 4.61E-06 | -0.06 | 0.07 | 0.34 |
| 9 |  | rs7712231 | A | G | 19.89 | 0.16 | 0.04 | 8.22E-06 | 0.06 | 0.10 | 0.53 |
| 10 |  | rs80309088 | G | A | 20.61 | 0.17 | 0.04 | 5.64E-06 | 0.19 | 0.10 | 0.05 |
| 11 |  | rs9887954 | G | A | 21.27 | -0.11 | 0.02 | 3.98E-06 | -0.07 | 0.07 | 0.28 |
| 1 | **Ruminiclostridium5** | rs10827477 | A | G | 22.59 | -0.05 | 0.01 | 2.00E-06 | 0.03 | 0.07 | 0.62 |
| 2 |  | rs113753996 | T | C | 22.13 | 0.08 | 0.02 | 2.55E-06 | -0.05 | 0.08 | 0.55 |
| 3 |  | rs1223978 | T | C | 20.00 | 0.05 | 0.01 | 7.73E-06 | -0.11 | 0.07 | 0.09 |
| 4 |  | rs1492620 | T | C | 21.27 | -0.08 | 0.02 | 3.99E-06 | 0.07 | 0.10 | 0.50 |
| 5 |  | rs2482038 | C | A | 22.76 | 0.05 | 0.01 | 1.83E-06 | -0.06 | 0.07 | 0.35 |
| 6 |  | rs2791343 | T | C | 20.81 | 0.05 | 0.01 | 5.07E-06 | -0.08 | 0.07 | 0.21 |
| 7 |  | rs2833828 | G | A | 20.29 | 0.05 | 0.01 | 6.66E-06 | 0.00 | 0.07 | 1.00 |
| 8 |  | rs4955951 | A | G | 18.53 | -0.07 | 0.02 | 1.68E-05 | 0.06 | 0.10 | 0.52 |
| 9 |  | rs6121460 | G | A | 21.93 | 0.09 | 0.02 | 2.82E-06 | -0.18 | 0.12 | 0.14 |
| 10 |  | rs79968837 | A | G | 24.12 | -0.10 | 0.02 | 9.06E-07 | 0.22 | 0.15 | 0.13 |
| 11 |  | rs8053158 | A | G | 21.65 | -0.07 | 0.02 | 3.27E-06 | -0.09 | 0.10 | 0.38 |
| 1 | **Ruminococcaceae (NK4A214group)** | rs11241747 | C | T | 19.78 | 0.05 | 0.01 | 8.69E-06 | -0.07 | 0.07 | 0.36 |
| 2 |  | rs11586410 | G | A | 25.81 | -0.09 | 0.02 | 3.76E-07 | 0.04 | 0.09 | 0.63 |
| 3 |  | rs12642039 | T | C | 21.45 | -0.06 | 0.01 | 3.63E-06 | 0.01 | 0.07 | 0.90 |
| 4 |  | rs12731 | A | G | 21.04 | -0.05 | 0.01 | 4.51E-06 | 0.11 | 0.07 | 0.12 |
| 5 |  | rs13087692 | T | G | 20.82 | 0.06 | 0.01 | 5.05E-06 | -0.09 | 0.07 | 0.22 |
| 6 |  | rs136761 | G | A | 24.31 | -0.06 | 0.01 | 8.19E-07 | 0.11 | 0.07 | 0.12 |
| 7 |  | rs147475196 | A | G | 20.53 | -0.13 | 0.03 | 5.86E-06 | -0.09 | 0.11 | 0.38 |
| 8 |  | rs35559912 | T | C | 20.63 | -0.09 | 0.02 | 5.57E-06 | 0.12 | 0.10 | 0.23 |
| 9 |  | rs4814689 | C | T | 22.04 | -0.11 | 0.02 | 2.68E-06 | 0.17 | 0.16 | 0.29 |
| 10 |  | rs5994253 | A | G | 26.49 | -0.08 | 0.02 | 2.64E-07 | 0.03 | 0.09 | 0.72 |
| 11 |  | rs62027366 | T | C | 20.00 | 0.06 | 0.01 | 7.75E-06 | -0.14 | 0.08 | 0.09 |
| 12 |  | rs6681678 | C | T | 17.42 | -0.10 | 0.02 | 2.99E-05 | -0.12 | 0.18 | 0.52 |
| 13 |  | rs7573569 | T | C | 21.26 | 0.11 | 0.02 | 4.00E-06 | 0.01 | 0.14 | 0.94 |
| 1 | **Ruminococcaceae UCG004** | rs10976229 | T | G | 20.01 | 0.10 | 0.02 | 7.70E-06 | 0.13 | 0.10 | 0.18 |
| 2 |  | rs11961899 | G | A | 19.24 | -0.07 | 0.02 | 1.15E-05 | 0.00 | 0.07 | 0.97 |
| 3 |  | rs12125734 | G | T | 27.09 | 0.13 | 0.03 | 1.95E-07 | 0.00 | 0.11 | 0.99 |
| 4 |  | rs2248146 | T | C | 20.13 | 0.07 | 0.02 | 7.22E-06 | 0.06 | 0.07 | 0.41 |
| 5 |  | rs3800178 | C | T | 20.44 | -0.08 | 0.02 | 6.15E-06 | -0.12 | 0.07 | 0.10 |
| 6 |  | rs516741 | T | G | 21.76 | -0.08 | 0.02 | 3.09E-06 | 0.00 | 0.08 | 0.95 |
| 7 |  | rs550351 | A | C | 19.06 | 0.08 | 0.02 | 1.27E-05 | 0.04 | 0.07 | 0.55 |
| 8 |  | rs6769553 | A | G | 29.13 | 0.08 | 0.02 | 6.78E-08 | 0.10 | 0.07 | 0.17 |
| 9 |  | rs7569771 | A | G | 19.87 | -0.08 | 0.02 | 8.29E-06 | -0.03 | 0.08 | 0.74 |
| 10 |  | rs872501 | G | A | 20.03 | 0.12 | 0.03 | 7.62E-06 | 0.02 | 0.12 | 0.87 |
| 11 |  | rs9818949 | G | T | 20.77 | 0.09 | 0.02 | 5.17E-06 | 0.04 | 0.08 | 0.63 |
| 1 | **Ruminococcaceae UCG005** | rs10937802 | G | A | 20.19 | 0.08 | 0.02 | 7.03E-06 | -0.08 | 0.10 | 0.43 |
| 2 |  | rs10950694 | T | C | 25.62 | 0.06 | 0.01 | 4.16E-07 | 0.19 | 0.07 | 0.01 |
| 3 |  | rs114279581 | A | G | 21.52 | -0.15 | 0.03 | 3.50E-06 | 0.03 | 0.12 | 0.84 |
| 4 |  | rs12288512 | A | G | 21.31 | 0.07 | 0.01 | 3.91E-06 | 0.11 | 0.08 | 0.14 |
| 5 |  | rs12604884 | G | T | 22.07 | 0.07 | 0.01 | 2.63E-06 | 0.00 | 0.08 | 0.99 |
| 6 |  | rs2893871 | G | A | 22.44 | -0.07 | 0.02 | 2.17E-06 | -0.30 | 0.11 | 0.00 |
| 7 |  | rs34781347 | G | A | 23.83 | 0.19 | 0.04 | 1.05E-06 | 0.16 | 0.13 | 0.21 |
| 8 |  | rs35166120 | C | G | 22.01 | -0.07 | 0.01 | 2.71E-06 | 0.13 | 0.08 | 0.09 |
| 9 |  | rs394449 | A | T | 21.71 | 0.07 | 0.01 | 3.17E-06 | -0.09 | 0.10 | 0.34 |
| 10 |  | rs72776570 | C | A | 19.50 | 0.09 | 0.02 | 1.01E-05 | 0.05 | 0.11 | 0.66 |
| 11 |  | rs7449320 | C | A | 20.97 | 0.06 | 0.01 | 4.66E-06 | -0.04 | 0.08 | 0.59 |
| 12 |  | rs7555878 | A | G | 21.93 | 0.06 | 0.01 | 2.83E-06 | 0.06 | 0.08 | 0.42 |
| 13 |  | rs7586445 | G | A | 19.66 | 0.08 | 0.02 | 9.27E-06 | 0.02 | 0.10 | 0.86 |
| 14 |  | rs898577 | T | C | 18.41 | -0.12 | 0.03 | 1.78E-05 | -0.02 | 0.14 | 0.89 |
| 1 | **Ruminococcus (gnavus group)** | rs11597105 | A | G | 20.93 | 0.11 | 0.03 | 4.76E-06 | -0.09 | 0.08 | 0.25 |
| 2 |  | rs11864644 | T | C | 19.30 | -0.14 | 0.03 | 1.12E-05 | 0.11 | 0.10 | 0.30 |
| 3 |  | rs12136548 | C | T | 21.07 | 0.09 | 0.02 | 4.42E-06 | -0.14 | 0.07 | 0.06 |
| 4 |  | rs12989336 | G | A | 20.31 | -0.08 | 0.02 | 6.60E-06 | -0.06 | 0.07 | 0.39 |
| 5 |  | rs13163520 | G | A | 29.66 | -0.13 | 0.02 | 5.15E-08 | 0.13 | 0.08 | 0.12 |
| 6 |  | rs2909242 | C | A | 24.59 | -0.09 | 0.02 | 7.10E-07 | 0.10 | 0.07 | 0.17 |
| 7 |  | rs3124783 | A | G | 21.68 | -0.12 | 0.02 | 3.22E-06 | 0.14 | 0.10 | 0.14 |
| 8 |  | rs4388134 | C | T | 19.77 | -0.09 | 0.02 | 8.74E-06 | -0.05 | 0.07 | 0.50 |
| 9 |  | rs62167033 | T | C | 21.86 | 0.19 | 0.04 | 2.93E-06 | -0.03 | 0.16 | 0.84 |
| 10 |  | rs78399089 | T | C | 19.56 | 0.14 | 0.03 | 9.76E-06 | 0.01 | 0.10 | 0.91 |
| 11 |  | rs934940 | A | C | 20.93 | -0.11 | 0.02 | 4.75E-06 | 0.04 | 0.10 | 0.71 |
| **Membranous nephropathy** | |  |  |  |  |  |  |  |  |  |  |
| 1 | **Butyricicoccus** | rs10084203 | A | G | 19.79 | 0.05 | 0.01 | 8.64E-06 | 0.06 | 0.05 | 0.24 |
| 2 |  | rs12034718 | A | G | 19.64 | 0.07 | 0.02 | 9.34E-06 | 0.09 | 0.06 | 0.16 |
| 3 |  | rs2017189 | G | T | 21.15 | -0.05 | 0.01 | 4.25E-06 | -0.01 | 0.04 | 0.83 |
| 4 |  | rs4962426 | G | T | 20.40 | 0.06 | 0.01 | 6.27E-06 | 0.06 | 0.05 | 0.28 |
| 5 |  | rs7322368 | T | C | 19.83 | 0.08 | 0.02 | 8.45E-06 | 0.04 | 0.07 | 0.58 |
| 1 | **Butyrivibrio** | rs1007475 | G | T | 20.42 | 0.12 | 0.03 | 6.22E-06 | 0.02 | 0.05 | 0.62 |
| 2 |  | rs11761679 | T | C | 23.17 | 0.15 | 0.03 | 1.48E-06 | 0.09 | 0.06 | 0.12 |
| 3 |  | rs17163238 | G | A | 20.80 | 0.14 | 0.03 | 5.09E-06 | 0.01 | 0.05 | 0.90 |
| 4 |  | rs28846706 | A | G | 20.16 | 0.18 | 0.04 | 7.13E-06 | 0.02 | 0.07 | 0.80 |
| 5 |  | rs4537857 | T | C | 22.79 | -0.12 | 0.03 | 1.81E-06 | -0.08 | 0.05 | 0.07 |
| 6 |  | rs486484 | A | G | 20.38 | -0.11 | 0.02 | 6.35E-06 | 0.05 | 0.04 | 0.22 |
| 7 |  | rs4928024 | A | G | 20.14 | -0.17 | 0.04 | 7.21E-06 | 0.05 | 0.07 | 0.48 |
| 8 |  | rs74622183 | A | G | 22.04 | -0.20 | 0.04 | 2.67E-06 | -0.07 | 0.07 | 0.32 |
| 9 |  | rs7752361 | A | G | 24.69 | -0.12 | 0.02 | 6.75E-07 | -0.02 | 0.04 | 0.56 |
| 10 |  | rs7763512 | G | A | 22.37 | 0.12 | 0.03 | 2.24E-06 | 0.05 | 0.05 | 0.27 |
| 11 |  | rs9349693 | A | G | 20.61 | 0.12 | 0.03 | 5.63E-06 | 0.06 | 0.04 | 0.16 |
| 1 | **Catenibacterium** | rs12404911 | C | T | 21.41 | 0.14 | 0.03 | 3.72E-06 | 0.03 | 0.05 | 0.59 |
| 2 |  | rs212393 | G | A | 22.33 | -0.14 | 0.03 | 2.29E-06 | -0.06 | 0.05 | 0.22 |
| 3 |  | rs73128290 | A | G | 20.78 | 0.13 | 0.03 | 5.14E-06 | 0.08 | 0.05 | 0.08 |
| 4 |  | rs7742829 | C | T | 20.65 | 0.11 | 0.03 | 5.51E-06 | 0.03 | 0.04 | 0.41 |
| 1 | **Oscillibacter** | rs11627628 | T | C | 24.61 | 0.14 | 0.03 | 7.04E-07 | 0.04 | 0.08 | 0.62 |
| 2 |  | rs11990279 | T | C | 20.90 | -0.08 | 0.02 | 4.85E-06 | 0.06 | 0.05 | 0.17 |
| 3 |  | rs12649930 | T | G | 21.94 | 0.12 | 0.03 | 2.82E-06 | 0.07 | 0.07 | 0.31 |
| 4 |  | rs16866406 | A | G | 22.43 | 0.10 | 0.02 | 2.18E-06 | -0.08 | 0.06 | 0.16 |
| 5 |  | rs16934185 | A | G | 21.17 | -0.13 | 0.03 | 4.19E-06 | 0.11 | 0.08 | 0.13 |
| 6 |  | rs36095275 | C | T | 23.01 | -0.08 | 0.02 | 1.62E-06 | 0.04 | 0.04 | 0.31 |
| 7 |  | rs61883564 | A | G | 21.03 | -0.10 | 0.02 | 4.52E-06 | 0.17 | 0.06 | 0.01 |
| 8 |  | rs62206502 | C | A | 20.27 | -0.07 | 0.02 | 6.72E-06 | -0.01 | 0.04 | 0.77 |
| 9 |  | rs9393920 | A | G | 24.29 | -0.07 | 0.02 | 8.27E-07 | 0.16 | 0.04 | 0.00 |
| 1 | **Ruminiclostridium5** | rs10827477 | A | G | 22.59 | -0.05 | 0.01 | 2.00E-06 | -0.02 | 0.05 | 0.72 |
| 2 |  | rs113753996 | T | C | 22.13 | 0.08 | 0.02 | 2.55E-06 | 0.05 | 0.07 | 0.45 |
| 3 |  | rs1223978 | T | C | 20.00 | 0.05 | 0.01 | 7.73E-06 | -0.03 | 0.04 | 0.52 |
| 4 |  | rs1492620 | T | C | 21.27 | -0.08 | 0.02 | 3.99E-06 | -0.04 | 0.07 | 0.61 |
| 5 |  | rs2482038 | C | A | 22.76 | 0.05 | 0.01 | 1.83E-06 | 0.06 | 0.04 | 0.13 |
| 6 |  | rs2791343 | T | C | 20.81 | 0.05 | 0.01 | 5.07E-06 | 0.00 | 0.04 | 0.99 |
| 7 |  | rs2801960 | C | G | 20.48 | 0.05 | 0.01 | 6.02E-06 | -0.03 | 0.04 | 0.51 |
| 8 |  | rs2833828 | G | A | 20.29 | 0.05 | 0.01 | 6.66E-06 | 0.03 | 0.04 | 0.54 |
| 9 |  | rs4955951 | A | G | 18.53 | -0.07 | 0.02 | 1.68E-05 | -0.02 | 0.07 | 0.74 |
| 10 |  | rs79968837 | A | G | 24.12 | -0.10 | 0.02 | 9.06E-07 | -0.07 | 0.08 | 0.37 |
| 11 |  | rs8053158 | A | G | 21.65 | -0.07 | 0.02 | 3.27E-06 | -0.15 | 0.06 | 0.01 |
| 1 | **Ruminococcaceae UCG003** | rs10490280 | C | T | 21.99 | -0.07 | 0.01 | 2.73E-06 | -0.08 | 0.05 | 0.10 |
| 2 |  | rs11243416 | T | C | 23.44 | -0.09 | 0.02 | 1.29E-06 | 0.02 | 0.07 | 0.76 |
| 3 |  | rs16959793 | A | C | 22.69 | -0.06 | 0.01 | 1.90E-06 | 0.01 | 0.05 | 0.84 |
| 4 |  | rs2523124 | T | C | 20.47 | -0.05 | 0.01 | 6.06E-06 | -0.01 | 0.04 | 0.89 |
| 5 |  | rs3013089 | G | A | 21.00 | -0.06 | 0.01 | 4.60E-06 | -0.09 | 0.04 | 0.03 |
| 6 |  | rs4452755 | A | C | 22.17 | -0.06 | 0.01 | 2.49E-06 | -0.02 | 0.05 | 0.73 |
| 7 |  | rs4532474 | G | A | 20.37 | 0.08 | 0.02 | 6.39E-06 | 0.04 | 0.06 | 0.46 |
| 8 |  | rs646327 | G | A | 24.57 | 0.06 | 0.01 | 7.18E-07 | 0.04 | 0.04 | 0.36 |
| 9 |  | rs6759615 | A | G | 26.22 | 0.10 | 0.02 | 3.04E-07 | 0.14 | 0.08 | 0.08 |
| 10 |  | rs73341548 | T | G | 28.47 | -0.17 | 0.03 | 9.50E-08 | -0.08 | 0.09 | 0.39 |
| 1 | **Ruminococcaceae UCG013** | rs12189346 | G | A | 22.14 | 0.07 | 0.01 | 2.54E-06 | -0.07 | 0.06 | 0.24 |
| 2 |  | rs12336782 | T | C | 20.45 | -0.09 | 0.02 | 6.13E-06 | 0.07 | 0.07 | 0.31 |
| 3 |  | rs12485353 | G | A | 21.58 | -0.06 | 0.01 | 3.39E-06 | 0.08 | 0.05 | 0.12 |
| 4 |  | rs12781711 | C | T | 31.19 | -0.07 | 0.01 | 2.33E-08 | 0.03 | 0.04 | 0.47 |
| 5 |  | rs16918863 | A | C | 21.55 | 0.11 | 0.02 | 3.44E-06 | 0.00 | 0.08 | 0.97 |
| 6 |  | rs2730183 | G | A | 19.77 | -0.05 | 0.01 | 8.73E-06 | 0.10 | 0.04 | 0.01 |
| 7 |  | rs4385846 | G | T | 20.61 | 0.06 | 0.01 | 5.63E-06 | 0.06 | 0.05 | 0.24 |
| 8 |  | rs75088940 | T | C | 22.07 | -0.09 | 0.02 | 2.63E-06 | 0.03 | 0.07 | 0.65 |
| **Glomerulonephritis** | |  |  |  |  |  |  |  |  |  |  |
| 1 | **Coprococcus3** | rs10810043 | A | G | 19.78 | 0.05 | 0.01 | 8.70E-06 | 0.06 | 0.02 | 0.01 |
| 2 |  | rs11077359 | T | C | 18.81 | -0.06 | 0.01 | 1.45E-05 | -0.03 | 0.03 | 0.38 |
| 3 |  | rs11080344 | C | T | 20.91 | 0.05 | 0.01 | 4.82E-06 | -0.01 | 0.02 | 0.76 |
| 4 |  | rs13247359 | G | A | 20.53 | 0.05 | 0.01 | 5.88E-06 | 0.01 | 0.02 | 0.78 |
| 5 |  | rs13394391 | C | T | 22.09 | -0.07 | 0.02 | 2.60E-06 | -0.03 | 0.03 | 0.30 |
| 6 |  | rs178271 | T | C | 24.37 | 0.15 | 0.03 | 7.95E-07 | -0.07 | 0.08 | 0.43 |
| 7 |  | rs4575475 | G | A | 20.22 | 0.06 | 0.01 | 6.92E-06 | 0.03 | 0.03 | 0.28 |
| 8 |  | rs7521171 | G | A | 21.28 | -0.06 | 0.01 | 3.96E-06 | -0.01 | 0.02 | 0.70 |
| 9 |  | rs8100692 | T | C | 25.89 | 0.06 | 0.01 | 3.61E-07 | 0.02 | 0.02 | 0.39 |
| 1 | **Erysipelotrichaceae UCG003** | rs11666127 | A | G | 19.86 | -0.07 | 0.02 | 8.32E-06 | 0.01 | 0.03 | 0.73 |
| 2 |  | rs11994308 | C | T | 22.65 | 0.12 | 0.02 | 1.94E-06 | -0.06 | 0.04 | 0.11 |
| 3 |  | rs12251396 | A | G | 19.74 | -0.07 | 0.02 | 8.87E-06 | -0.01 | 0.03 | 0.70 |
| 4 |  | rs17798136 | G | A | 20.87 | 0.16 | 0.03 | 4.92E-06 | -0.02 | 0.04 | 0.58 |
| 5 |  | rs2044550 | T | C | 20.06 | -0.06 | 0.01 | 7.50E-06 | -0.01 | 0.03 | 0.73 |
| 6 |  | rs28568391 | A | G | 24.18 | -0.06 | 0.01 | 8.76E-07 | 0.03 | 0.02 | 0.14 |
| 7 |  | rs4758231 | G | T | 20.44 | -0.06 | 0.01 | 6.16E-06 | 0.02 | 0.02 | 0.39 |
| 8 |  | rs59068084 | T | G | 22.07 | 0.06 | 0.01 | 2.63E-06 | 0.00 | 0.02 | 0.92 |
| 9 |  | rs62403464 | T | C | 21.86 | -0.07 | 0.02 | 2.94E-06 | 0.04 | 0.03 | 0.13 |
| 10 |  | rs6875357 | C | T | 21.92 | 0.17 | 0.04 | 2.84E-06 | -0.05 | 0.06 | 0.38 |
| 11 |  | rs73074432 | C | T | 19.27 | 0.07 | 0.02 | 1.14E-05 | -0.06 | 0.03 | 0.10 |
| 12 |  | rs75949021 | T | C | 20.55 | -0.17 | 0.04 | 5.80E-06 | 0.14 | 0.06 | 0.01 |
| 13 |  | rs76502207 | T | C | 24.96 | 0.14 | 0.03 | 5.86E-07 | 0.05 | 0.06 | 0.40 |
| 14 |  | rs8053479 | A | G | 20.20 | -0.08 | 0.02 | 6.98E-06 | 0.02 | 0.03 | 0.60 |
| 1 | **Oxalobacter** | rs10464997 | G | A | 21.81 | 0.14 | 0.03 | 3.00E-06 | 0.06 | 0.03 | 0.05 |
| 2 |  | rs11108500 | A | G | 21.71 | -0.20 | 0.04 | 3.17E-06 | 0.00 | 0.04 | 0.94 |
| 3 |  | rs111966731 | T | C | 20.42 | 0.21 | 0.05 | 6.22E-06 | 0.02 | 0.04 | 0.65 |
| 4 |  | rs12002250 | A | C | 21.68 | 0.22 | 0.05 | 3.22E-06 | 0.01 | 0.05 | 0.84 |
| 5 |  | rs1569853 | T | C | 21.62 | -0.14 | 0.03 | 3.33E-06 | -0.02 | 0.03 | 0.61 |
| 6 |  | rs36057338 | G | T | 24.32 | 0.21 | 0.04 | 8.15E-07 | 0.05 | 0.06 | 0.44 |
| 7 |  | rs3862635 | C | T | 19.09 | -0.17 | 0.04 | 1.25E-05 | -0.03 | 0.04 | 0.49 |
| 8 |  | rs4428215 | G | A | 28.93 | 0.13 | 0.02 | 7.50E-08 | 0.04 | 0.03 | 0.14 |
| 9 |  | rs6000536 | C | T | 26.64 | -0.13 | 0.03 | 2.45E-07 | -0.01 | 0.03 | 0.86 |
| 10 |  | rs6993398 | G | A | 20.81 | 0.13 | 0.03 | 5.06E-06 | 0.04 | 0.03 | 0.12 |
| 11 |  | rs736744 | C | T | 31.14 | 0.12 | 0.02 | 2.41E-08 | 0.04 | 0.02 | 0.05 |
| **Acute tubulo-interstitial nephritis** | | |  |  |  |  |  |  |  |  |  |
| 1 | **Actinomyces** | rs2715439 | C | T | 20.52 | 0.07 | 0.02 | 5.89E-06 | -0.01 | 0.01 | 0.58 |
| 2 |  | rs34583783 | G | T | 22.24 | 0.13 | 0.03 | 2.41E-06 | -0.02 | 0.03 | 0.55 |
| 3 |  | rs35011108 | A | G | 20.64 | 0.23 | 0.05 | 5.54E-06 | -0.05 | 0.03 | 0.06 |
| 4 |  | rs4073240 | G | A | 20.06 | 0.07 | 0.02 | 7.49E-06 | -0.01 | 0.01 | 0.72 |
| 5 |  | rs4146653 | G | A | 21.16 | 0.10 | 0.02 | 4.23E-06 | -0.03 | 0.02 | 0.09 |
| 6 |  | rs71315246 | A | G | 19.57 | -0.10 | 0.02 | 9.72E-06 | 0.01 | 0.02 | 0.58 |
| 7 |  | rs7915461 | T | C | 21.85 | 0.19 | 0.04 | 2.94E-06 | -0.03 | 0.03 | 0.32 |
| 1 | **Gordonibacter** | rs13412653 | A | C | 20.23 | 0.11 | 0.02 | 6.87E-06 | 0.01 | 0.01 | 0.58 |
| 2 |  | rs16955299 | G | A | 20.53 | -0.20 | 0.04 | 5.88E-06 | 0.03 | 0.02 | 0.26 |
| 3 |  | rs322296 | G | A | 22.44 | 0.18 | 0.04 | 2.17E-06 | -0.01 | 0.03 | 0.82 |
| 4 |  | rs35042269 | C | A | 19.99 | -0.18 | 0.04 | 7.80E-06 | 0.07 | 0.02 | 0.00 |
| 5 |  | rs3765837 | T | G | 19.35 | -0.19 | 0.04 | 1.09E-05 | 0.03 | 0.03 | 0.24 |
| 6 |  | rs4596722 | A | G | 19.75 | 0.10 | 0.02 | 8.83E-06 | 0.00 | 0.01 | 0.82 |
| 7 |  | rs72714787 | C | A | 23.14 | 0.18 | 0.04 | 1.51E-06 | -0.03 | 0.02 | 0.17 |
| 8 |  | rs72939513 | A | G | 19.03 | -0.21 | 0.05 | 1.29E-05 | -0.01 | 0.03 | 0.80 |
| 9 |  | rs7294633 | C | T | 26.50 | 0.13 | 0.02 | 2.63E-07 | 0.00 | 0.02 | 0.88 |
| 10 |  | rs768830 | G | A | 20.21 | 0.15 | 0.03 | 6.93E-06 | -0.03 | 0.02 | 0.19 |
| 1 | **Marvinbryantia** | rs11620597 | T | C | 19.34 | 0.12 | 0.03 | 1.09E-05 | 0.13 | 0.05 | 0.00 |
| 2 |  | rs1187983 | C | T | 23.45 | -0.09 | 0.02 | 1.28E-06 | -0.03 | 0.02 | 0.28 |
| 3 |  | rs146541147 | G | A | 19.60 | 0.12 | 0.03 | 9.53E-06 | 0.03 | 0.04 | 0.43 |
| 4 |  | rs2724813 | A | G | 25.18 | -0.08 | 0.02 | 5.22E-07 | -0.03 | 0.02 | 0.10 |
| 5 |  | rs2842896 | C | T | 24.52 | -0.06 | 0.01 | 7.36E-07 | -0.02 | 0.01 | 0.14 |
| 6 |  | rs2863363 | A | G | 21.69 | 0.06 | 0.01 | 3.21E-06 | 0.03 | 0.02 | 0.08 |
| 7 |  | rs3125832 | A | C | 20.48 | 0.07 | 0.02 | 6.03E-06 | 0.01 | 0.02 | 0.62 |
| 8 |  | rs61884471 | G | A | 25.08 | 0.12 | 0.02 | 5.49E-07 | 0.00 | 0.02 | 0.94 |
| 9 |  | rs72948274 | A | C | 21.55 | -0.13 | 0.03 | 3.45E-06 | -0.06 | 0.03 | 0.04 |
| 10 |  | rs8006832 | G | T | 19.32 | -0.10 | 0.02 | 1.11E-05 | 0.00 | 0.02 | 0.91 |
| 1 | **Odoribacter** | rs10093869 | A | G | 21.23 | -0.06 | 0.01 | 4.07E-06 | -0.02 | 0.01 | 0.25 |
| 2 |  | rs10423795 | C | T | 20.66 | 0.06 | 0.01 | 5.49E-06 | 0.02 | 0.01 | 0.18 |
| 3 |  | rs28417404 | A | G | 20.29 | -0.07 | 0.02 | 6.65E-06 | -0.02 | 0.02 | 0.37 |
| 4 |  | rs4793970 | A | G | 19.91 | -0.06 | 0.01 | 8.11E-06 | -0.03 | 0.01 | 0.08 |
| 5 |  | rs6856150 | G | A | 20.63 | 0.09 | 0.02 | 5.56E-06 | 0.02 | 0.02 | 0.28 |
| 6 |  | rs74553962 | T | G | 21.15 | 0.12 | 0.03 | 4.26E-06 | 0.03 | 0.03 | 0.20 |
| 7 |  | rs77779484 | G | A | 24.71 | -0.13 | 0.03 | 6.65E-07 | 0.03 | 0.03 | 0.23 |
| **Chronic tubulo-interstitial nephritis** | | |  |  |  |  |  |  |  |  |  |
| 1 | **Coprococcus3** | rs10810043 | A | G | 19.78 | 0.05 | 0.01 | 8.70E-06 | 0.11 | 0.06 | 0.07 |
| 2 |  | rs11077359 | T | C | 18.81 | -0.06 | 0.01 | 1.45E-05 | -0.14 | 0.08 | 0.08 |
| 3 |  | rs11080344 | C | T | 20.91 | 0.05 | 0.01 | 4.82E-06 | 0.06 | 0.06 | 0.29 |
| 4 |  | rs13247359 | G | A | 20.53 | 0.05 | 0.01 | 5.88E-06 | -0.01 | 0.06 | 0.82 |
| 5 |  | rs13394391 | C | T | 22.09 | -0.07 | 0.02 | 2.60E-06 | -0.14 | 0.08 | 0.07 |
| 6 |  | rs178271 | T | C | 24.37 | 0.15 | 0.03 | 7.95E-07 | -0.27 | 0.22 | 0.22 |
| 7 |  | rs4575475 | G | A | 20.22 | 0.06 | 0.01 | 6.92E-06 | 0.01 | 0.07 | 0.91 |
| 8 |  | rs7521171 | G | A | 21.28 | -0.06 | 0.01 | 3.96E-06 | -0.07 | 0.06 | 0.24 |
| 9 |  | rs8100692 | T | C | 25.89 | 0.06 | 0.01 | 3.61E-07 | 0.09 | 0.06 | 0.10 |
| 1 | **Dorea** | rs11150408 | T | G | 20.04 | 0.05 | 0.01 | 7.58E-06 | -0.10 | 0.06 | 0.08 |
| 2 |  | rs12537781 | T | C | 19.67 | -0.06 | 0.01 | 9.18E-06 | -0.01 | 0.07 | 0.89 |
| 3 |  | rs13279148 | G | A | 22.48 | 0.07 | 0.02 | 2.13E-06 | -0.09 | 0.09 | 0.30 |
| 4 |  | rs1899291 | C | T | 21.52 | 0.07 | 0.02 | 3.50E-06 | -0.14 | 0.08 | 0.07 |
| 5 |  | rs3005511 | A | G | 20.92 | 0.05 | 0.01 | 4.80E-06 | 0.00 | 0.06 | 0.96 |
| 6 |  | rs345219 | T | G | 19.50 | -0.05 | 0.01 | 1.00E-05 | 0.00 | 0.06 | 0.94 |
| 7 |  | rs3752849 | G | A | 20.03 | 0.16 | 0.04 | 7.62E-06 | -0.09 | 0.13 | 0.49 |
| 8 |  | rs4793307 | C | T | 21.98 | 0.06 | 0.01 | 2.76E-06 | -0.05 | 0.07 | 0.44 |
| 9 |  | rs62503162 | A | G | 25.11 | -0.10 | 0.02 | 5.41E-07 | 0.03 | 0.14 | 0.81 |
| 10 |  | rs73729431 | C | T | 20.99 | -0.14 | 0.03 | 4.61E-06 | 0.11 | 0.20 | 0.58 |
| 1 | **Erysipelotrichaceae UCG003** | rs11666127 | A | G | 19.86 | -0.07 | 0.02 | 8.32E-06 | -0.03 | 0.08 | 0.73 |
| 2 |  | rs11994308 | C | T | 22.65 | 0.12 | 0.02 | 1.94E-06 | -0.15 | 0.10 | 0.14 |
| 3 |  | rs12251396 | A | G | 19.74 | -0.07 | 0.02 | 8.87E-06 | 0.02 | 0.08 | 0.78 |
| 4 |  | rs17798136 | G | A | 20.87 | 0.16 | 0.03 | 4.92E-06 | -0.10 | 0.12 | 0.38 |
| 5 |  | rs2044550 | T | C | 20.06 | -0.06 | 0.01 | 7.50E-06 | 0.02 | 0.07 | 0.82 |
| 6 |  | rs28568391 | A | G | 24.18 | -0.06 | 0.01 | 8.76E-07 | 0.12 | 0.06 | 0.04 |
| 7 |  | rs4758231 | G | T | 20.44 | -0.06 | 0.01 | 6.16E-06 | 0.08 | 0.06 | 0.22 |
| 8 |  | rs59068084 | T | G | 22.07 | 0.06 | 0.01 | 2.63E-06 | 0.01 | 0.06 | 0.91 |
| 9 |  | rs62403464 | T | C | 21.86 | -0.07 | 0.02 | 2.94E-06 | -0.05 | 0.07 | 0.48 |
| 10 |  | rs6875357 | C | T | 21.92 | 0.17 | 0.04 | 2.84E-06 | 0.01 | 0.15 | 0.95 |
| 11 |  | rs73074432 | C | T | 19.27 | 0.07 | 0.02 | 1.14E-05 | -0.14 | 0.09 | 0.14 |
| 12 |  | rs75949021 | T | C | 20.55 | -0.17 | 0.04 | 5.80E-06 | 0.09 | 0.15 | 0.53 |
| 13 |  | rs76502207 | T | C | 24.96 | 0.14 | 0.03 | 5.86E-07 | -0.22 | 0.15 | 0.13 |
| 14 |  | rs8053479 | A | G | 20.20 | -0.08 | 0.02 | 6.98E-06 | 0.05 | 0.09 | 0.59 |
| 1 | **Eubacterium (brachy group)** | rs12151423 | A | G | 19.87 | 0.10 | 0.02 | 8.29E-06 | -0.07 | 0.06 | 0.25 |
| 2 |  | rs13139592 | T | C | 19.91 | -0.15 | 0.03 | 8.12E-06 | 0.17 | 0.08 | 0.04 |
| 3 |  | rs1384962 | A | G | 20.61 | 0.12 | 0.03 | 5.62E-06 | 0.01 | 0.06 | 0.91 |
| 4 |  | rs2913110 | C | T | 21.00 | 0.11 | 0.02 | 4.58E-06 | -0.03 | 0.06 | 0.57 |
| 5 |  | rs4862235 | G | A | 21.55 | 0.10 | 0.02 | 3.44E-06 | -0.08 | 0.06 | 0.18 |
| 6 |  | rs55932844 | A | G | 22.36 | -0.17 | 0.04 | 2.26E-06 | 0.12 | 0.10 | 0.26 |
| 7 |  | rs62348779 | T | C | 21.67 | -0.20 | 0.04 | 3.25E-06 | -0.06 | 0.11 | 0.59 |
| 8 |  | rs6591893 | G | A | 20.28 | 0.11 | 0.02 | 6.69E-06 | 0.05 | 0.06 | 0.44 |
| 9 |  | rs720439 | A | G | 19.84 | -0.11 | 0.03 | 8.40E-06 | 0.10 | 0.07 | 0.15 |
| 10 |  | rs73199919 | T | C | 19.85 | -0.24 | 0.05 | 8.38E-06 | 0.04 | 0.13 | 0.78 |
| 1 | **Intestinimonas** | rs10262702 | T | C | 22.19 | 0.09 | 0.02 | 2.47E-06 | -0.01 | 0.09 | 0.90 |
| 2 |  | rs11258178 | A | G | 24.26 | 0.07 | 0.01 | 8.40E-07 | 0.02 | 0.06 | 0.71 |
| 3 |  | rs12226153 | A | G | 24.25 | -0.15 | 0.03 | 8.46E-07 | 0.12 | 0.24 | 0.63 |
| 4 |  | rs12566247 | T | A | 22.15 | 0.06 | 0.01 | 2.52E-06 | -0.07 | 0.06 | 0.21 |
| 5 |  | rs17067892 | C | T | 18.38 | 0.11 | 0.03 | 1.81E-05 | 0.01 | 0.10 | 0.91 |
| 6 |  | rs1859797 | G | A | 20.98 | 0.06 | 0.01 | 4.64E-06 | 0.05 | 0.06 | 0.38 |
| 7 |  | rs2276760 | A | G | 20.18 | -0.07 | 0.02 | 7.06E-06 | 0.03 | 0.07 | 0.64 |
| 8 |  | rs2731794 | C | T | 21.94 | 0.12 | 0.03 | 2.81E-06 | 0.13 | 0.16 | 0.39 |
| 9 |  | rs2930225 | G | T | 22.75 | 0.07 | 0.02 | 1.84E-06 | 0.05 | 0.07 | 0.45 |
| 10 |  | rs4113676 | A | C | 19.87 | -0.22 | 0.05 | 8.27E-06 | -0.01 | 0.24 | 0.95 |
| 11 |  | rs4784055 | T | C | 20.63 | -0.18 | 0.04 | 5.57E-06 | -0.26 | 0.13 | 0.05 |
| 12 |  | rs62240188 | G | A | 23.70 | 0.13 | 0.03 | 1.13E-06 | -0.02 | 0.10 | 0.87 |
| 13 |  | rs6934519 | C | T | 20.98 | 0.07 | 0.02 | 4.64E-06 | -0.05 | 0.07 | 0.49 |
| 14 |  | rs716604 | A | G | 24.29 | 0.08 | 0.02 | 8.29E-07 | 0.06 | 0.07 | 0.36 |
| 15 |  | rs7170984 | T | C | 21.86 | -0.07 | 0.01 | 2.94E-06 | -0.07 | 0.06 | 0.25 |
| 16 |  | rs72982915 | C | T | 20.68 | 0.18 | 0.04 | 5.42E-06 | 0.03 | 0.12 | 0.79 |
| 17 |  | rs9823439 | T | C | 19.60 | -0.06 | 0.01 | 9.56E-06 | -0.13 | 0.06 | 0.03 |
| 1 | **Victivallis** | rs11899949 | G | A | 22.34 | 0.13 | 0.03 | 2.28E-06 | 0.03 | 0.06 | 0.59 |
| 2 |  | rs12512543 | A | C | 22.62 | -0.18 | 0.04 | 1.98E-06 | 0.08 | 0.10 | 0.43 |
| 3 |  | rs173120 | T | C | 21.27 | 0.13 | 0.03 | 3.99E-06 | 0.06 | 0.07 | 0.45 |
| 4 |  | rs1882775 | A | G | 19.55 | -0.14 | 0.03 | 9.81E-06 | 0.08 | 0.07 | 0.31 |
| 5 |  | rs2546432 | T | C | 19.70 | -0.11 | 0.02 | 9.07E-06 | -0.07 | 0.06 | 0.20 |
| 6 |  | rs342302 | A | G | 18.88 | -0.15 | 0.04 | 1.39E-05 | -0.11 | 0.08 | 0.20 |
| 7 |  | rs4764863 | G | A | 24.41 | 0.12 | 0.02 | 7.80E-07 | 0.12 | 0.06 | 0.03 |
| 8 |  | rs4895919 | T | C | 22.31 | -0.12 | 0.02 | 2.32E-06 | -0.03 | 0.06 | 0.60 |
| 9 |  | rs56349194 | A | G | 25.28 | -0.16 | 0.03 | 4.95E-07 | -0.09 | 0.09 | 0.30 |
| 10 |  | rs911666 | T | C | 20.30 | -0.12 | 0.03 | 6.60E-06 | -0.06 | 0.06 | 0.33 |
| **Chronic kidney disease** | |  |  |  |  |  |  |  |  |  |  |
| 1 | **Butyrivibrio** | rs1007475 | G | T | 20.42 | 0.12 | 0.03 | 6.22E-06 | 0.01 | 0.03 | 0.69 |
| 2 |  | rs11761679 | T | C | 23.17 | 0.15 | 0.03 | 1.48E-06 | -0.01 | 0.03 | 0.75 |
| 3 |  | rs142855850 | A | G | 20.11 | 0.21 | 0.05 | 7.30E-06 | -0.02 | 0.04 | 0.58 |
| 4 |  | rs16934069 | T | C | 19.96 | -0.13 | 0.03 | 7.90E-06 | -0.05 | 0.03 | 0.13 |
| 5 |  | rs16941336 | C | T | 22.64 | 0.13 | 0.03 | 1.95E-06 | 0.02 | 0.03 | 0.45 |
| 6 |  | rs17163238 | G | A | 20.80 | 0.14 | 0.03 | 5.09E-06 | 0.02 | 0.03 | 0.54 |
| 7 |  | rs28846706 | A | G | 20.16 | 0.18 | 0.04 | 7.13E-06 | 0.02 | 0.04 | 0.65 |
| 8 |  | rs4537857 | T | C | 22.79 | -0.12 | 0.03 | 1.81E-06 | -0.02 | 0.03 | 0.40 |
| 9 |  | rs486484 | A | G | 20.38 | -0.11 | 0.02 | 6.35E-06 | -0.04 | 0.02 | 0.12 |
| 10 |  | rs4928024 | A | G | 20.14 | -0.17 | 0.04 | 7.21E-06 | -0.05 | 0.03 | 0.14 |
| 11 |  | rs72723662 | C | T | 24.87 | 0.22 | 0.04 | 6.14E-07 | -0.07 | 0.04 | 0.04 |
| 12 |  | rs74622183 | A | G | 22.04 | -0.20 | 0.04 | 2.67E-06 | -0.01 | 0.04 | 0.80 |
| 13 |  | rs7752361 | A | G | 24.69 | -0.12 | 0.02 | 6.75E-07 | -0.04 | 0.02 | 0.08 |
| 14 |  | rs7763512 | G | A | 22.37 | 0.12 | 0.03 | 2.24E-06 | 0.02 | 0.02 | 0.52 |
| 15 |  | rs9349693 | A | G | 20.61 | 0.12 | 0.03 | 5.63E-06 | 0.04 | 0.03 | 0.12 |
| 1 | **Coprococcus3** | rs10810043 | A | G | 19.78 | 0.05 | 0.01 | 8.70E-06 | 0.07 | 0.03 | 0.01 |
| 2 |  | rs11077359 | T | C | 18.81 | -0.06 | 0.01 | 1.45E-05 | -0.04 | 0.03 | 0.21 |
| 3 |  | rs11080344 | C | T | 20.91 | 0.05 | 0.01 | 4.82E-06 | 0.00 | 0.02 | 0.89 |
| 4 |  | rs13247359 | G | A | 20.53 | 0.05 | 0.01 | 5.88E-06 | -0.01 | 0.02 | 0.76 |
| 5 |  | rs13394391 | C | T | 22.09 | -0.07 | 0.02 | 2.60E-06 | -0.02 | 0.03 | 0.57 |
| 6 |  | rs178271 | T | C | 24.37 | 0.15 | 0.03 | 7.95E-07 | -0.04 | 0.09 | 0.64 |
| 7 |  | rs4575475 | G | A | 20.22 | 0.06 | 0.01 | 6.92E-06 | 0.04 | 0.03 | 0.12 |
| 8 |  | rs7521171 | G | A | 21.28 | -0.06 | 0.01 | 3.96E-06 | -0.01 | 0.03 | 0.81 |
| 9 |  | rs8100692 | T | C | 25.89 | 0.06 | 0.01 | 3.61E-07 | 0.01 | 0.02 | 0.58 |
| 1 | **Erysipelotrichaceae UCG003** | rs11666127 | A | G | 19.86 | -0.07 | 0.02 | 8.32E-06 | 0.00 | 0.03 | 0.96 |
| 2 |  | rs11994308 | C | T | 22.65 | 0.12 | 0.02 | 1.94E-06 | -0.04 | 0.04 | 0.30 |
| 3 |  | rs12251396 | A | G | 19.74 | -0.07 | 0.02 | 8.87E-06 | 0.00 | 0.03 | 0.95 |
| 4 |  | rs17798136 | G | A | 20.87 | 0.16 | 0.03 | 4.92E-06 | 0.01 | 0.05 | 0.81 |
| 5 |  | rs2044550 | T | C | 20.06 | -0.06 | 0.01 | 7.50E-06 | -0.01 | 0.03 | 0.68 |
| 6 |  | rs28568391 | A | G | 24.18 | -0.06 | 0.01 | 8.76E-07 | 0.05 | 0.02 | 0.06 |
| 7 |  | rs4758231 | G | T | 20.44 | -0.06 | 0.01 | 6.16E-06 | 0.04 | 0.03 | 0.09 |
| 8 |  | rs59068084 | T | G | 22.07 | 0.06 | 0.01 | 2.63E-06 | -0.02 | 0.02 | 0.33 |
| 9 |  | rs62403464 | T | C | 21.86 | -0.07 | 0.02 | 2.94E-06 | 0.05 | 0.03 | 0.12 |
| 10 |  | rs6875357 | C | T | 21.92 | 0.17 | 0.04 | 2.84E-06 | -0.07 | 0.06 | 0.24 |
| 11 |  | rs73074432 | C | T | 19.27 | 0.07 | 0.02 | 1.14E-05 | -0.03 | 0.04 | 0.40 |
| 12 |  | rs75949021 | T | C | 20.55 | -0.17 | 0.04 | 5.80E-06 | 0.15 | 0.06 | 0.01 |
| 13 |  | rs76502207 | T | C | 24.96 | 0.14 | 0.03 | 5.86E-07 | 0.01 | 0.06 | 0.85 |
| 14 |  | rs8053479 | A | G | 20.20 | -0.08 | 0.02 | 6.98E-06 | 0.01 | 0.04 | 0.87 |
| 1 | **Lachnospira** | rs13157098 | A | G | 24.46 | -0.08 | 0.02 | 7.60E-07 | 0.02 | 0.03 | 0.56 |
| 2 |  | rs2520509 | A | G | 20.10 | 0.05 | 0.01 | 7.35E-06 | 0.00 | 0.03 | 0.91 |
| 3 |  | rs4686798 | T | C | 21.86 | 0.05 | 0.01 | 2.93E-06 | -0.02 | 0.02 | 0.35 |
| 4 |  | rs4923324 | G | A | 21.42 | -0.06 | 0.01 | 3.69E-06 | 0.07 | 0.03 | 0.02 |
| 5 |  | rs56791201 | T | C | 21.92 | 0.05 | 0.01 | 2.85E-06 | -0.03 | 0.02 | 0.20 |
| 1 | **Oxalobacter** | rs10464997 | G | A | 21.81 | 0.14 | 0.03 | 3.00E-06 | 0.06 | 0.03 | 0.04 |
| 2 |  | rs11108500 | A | G | 21.71 | -0.20 | 0.04 | 3.17E-06 | -0.01 | 0.04 | 0.89 |
| 3 |  | rs111966731 | T | C | 20.42 | 0.21 | 0.05 | 6.22E-06 | -0.01 | 0.04 | 0.89 |
| 4 |  | rs12002250 | A | C | 21.68 | 0.22 | 0.05 | 3.22E-06 | -0.03 | 0.06 | 0.62 |
| 5 |  | rs1569853 | T | C | 21.62 | -0.14 | 0.03 | 3.33E-06 | -0.01 | 0.04 | 0.74 |
| 6 |  | rs36057338 | G | T | 24.32 | 0.21 | 0.04 | 8.15E-07 | 0.09 | 0.07 | 0.18 |
| 7 |  | rs3862635 | C | T | 19.09 | -0.17 | 0.04 | 1.25E-05 | -0.03 | 0.04 | 0.53 |
| 8 |  | rs4428215 | G | A | 28.93 | 0.13 | 0.02 | 7.50E-08 | 0.02 | 0.03 | 0.43 |
| 9 |  | rs6000536 | C | T | 26.64 | -0.13 | 0.03 | 2.45E-07 | 0.01 | 0.03 | 0.87 |
| 10 |  | rs6993398 | G | A | 20.81 | 0.13 | 0.03 | 5.06E-06 | 0.05 | 0.03 | 0.14 |
| 11 |  | rs736744 | C | T | 31.14 | 0.12 | 0.02 | 2.41E-08 | 0.05 | 0.02 | 0.03 |
| 1 | **Prevotella7** | rs11035469 | A | G | 21.36 | -0.14 | 0.03 | 3.80E-06 | 0.03 | 0.03 | 0.29 |
| 2 |  | rs118038478 | A | G | 19.24 | 0.21 | 0.05 | 1.15E-05 | 0.02 | 0.05 | 0.63 |
| 3 |  | rs12124567 | A | G | 19.45 | -0.12 | 0.03 | 1.03E-05 | -0.02 | 0.03 | 0.40 |
| 4 |  | rs12195431 | T | C | 19.74 | 0.20 | 0.04 | 8.87E-06 | 0.05 | 0.04 | 0.21 |
| 5 |  | rs2240542 | C | T | 21.31 | 0.12 | 0.03 | 3.90E-06 | 0.03 | 0.03 | 0.34 |
| 6 |  | rs2918132 | C | T | 20.23 | -0.11 | 0.03 | 6.88E-06 | -0.03 | 0.02 | 0.15 |
| 7 |  | rs385483 | A | G | 22.04 | 0.14 | 0.03 | 2.67E-06 | -0.01 | 0.03 | 0.79 |
| 8 |  | rs57404562 | C | A | 24.20 | 0.16 | 0.03 | 8.67E-07 | 0.05 | 0.04 | 0.14 |
| 9 |  | rs9426434 | T | C | 19.71 | -0.12 | 0.03 | 9.00E-06 | 0.01 | 0.03 | 0.61 |
| 10 |  | rs9608249 | A | G | 22.13 | -0.16 | 0.03 | 2.55E-06 | -0.04 | 0.04 | 0.34 |
| 11 |  | rs9959718 | G | A | 23.33 | 0.13 | 0.03 | 1.36E-06 | 0.07 | 0.03 | 0.01 |
| 1 | **Ruminococcus2** | rs12406309 | A | C | 19.73 | -0.06 | 0.01 | 8.93E-06 | -0.03 | 0.03 | 0.33 |
| 2 |  | rs1819812 | G | T | 20.76 | 0.08 | 0.02 | 5.22E-06 | -0.03 | 0.05 | 0.59 |
| 3 |  | rs2368224 | T | G | 20.73 | 0.20 | 0.04 | 5.28E-06 | 0.12 | 0.05 | 0.02 |
| 4 |  | rs2846589 | G | T | 20.12 | 0.05 | 0.01 | 7.26E-06 | -0.01 | 0.02 | 0.82 |
| 5 |  | rs2997412 | A | G | 21.59 | -0.06 | 0.01 | 3.37E-06 | -0.03 | 0.03 | 0.25 |
| 6 |  | rs4400279 | A | G | 20.68 | 0.05 | 0.01 | 5.42E-06 | 0.03 | 0.03 | 0.18 |
| 7 |  | rs4799823 | C | T | 21.09 | 0.08 | 0.02 | 4.38E-06 | 0.03 | 0.03 | 0.26 |
| 8 |  | rs55707116 | C | A | 20.94 | 0.09 | 0.02 | 4.74E-06 | 0.02 | 0.04 | 0.71 |
| 9 |  | rs58681734 | A | G | 20.27 | 0.07 | 0.02 | 6.73E-06 | -0.04 | 0.03 | 0.15 |
| 10 |  | rs61791565 | T | C | 19.98 | -0.05 | 0.01 | 7.83E-06 | -0.01 | 0.02 | 0.54 |
| 11 |  | rs75140805 | T | G | 22.49 | 0.08 | 0.02 | 2.11E-06 | 0.04 | 0.03 | 0.24 |
| 12 |  | rs7635831 | G | A | 22.97 | 0.06 | 0.01 | 1.65E-06 | 0.04 | 0.02 | 0.13 |
| 13 |  | rs7693984 | G | A | 19.13 | -0.10 | 0.02 | 1.22E-05 | 0.06 | 0.06 | 0.33 |
| 14 |  | rs78120384 | A | G | 24.19 | -0.19 | 0.04 | 8.73E-07 | 0.00 | 0.04 | 0.94 |

MR = mendelian randomization; SNPs = single nucleotide polymorphisms.

**Supplemental Table 3. Heterogeneity in instrumental variables for gut microbiota**

| **Kidney disease** | **Bacterial genus(exposure)** | **Cochran's Q** | **df** | **P-value** |
| --- | --- | --- | --- | --- |
| **Nephrotic syndrome** | Akkermansia | 7.83 | 10 | 0.65 |
|  | Bacteroides | 6.82 | 6 | 0.34 |
|  | Christensenellaceae (R-7 group) | 6.32 | 7 | 0.50 |
|  | Gordonibacter | 6.45 | 9 | 0.69 |
|  | Lachnospiraceae (ND3007 group) | 2.29 | 2 | 0.32 |
|  | Oxalobacter | 4.98 | 10 | 0.89 |
|  | Rikenellaceae RC9 (gut group) | 7.01 | 10 | 0.72 |
|  | Ruminiclostridium5 | 5.75 | 10 | 0.84 |
|  | Ruminococcaceae (NK4A214 group) | 9.43 | 12 | 0.67 |
|  | Ruminococcaceae UCG004 | 3.41 | 10 | 0.97 |
|  | Ruminococcaceae UCG005 | 16.10 | 13 | 0.24 |
|  | Ruminococcus (gnavus group) | 8.47 | 10 | 0.58 |
| **Membranous nephropathy** | Butyricicoccus | 0.88 | 3 | 0.83 |
|  | Butyrivibrio | 8.58 | 10 | 0.57 |
|  | Catenibacterium | 0.75 | 3 | 0.86 |
|  | Oscillibacter | 20.11 | 8 | 0.01 |
|  | Ruminiclostridium5 | 6.68 | 10 | 0.76 |
|  | Ruminococcaceae UCG003 | 6.63 | 9 | 0.68 |
|  | Ruminococcaceae UCG013 | 9.02 | 8 | 0.34 |
| **Glomerulonephritis** | Coprococcus3 | 7.83 | 8 | 0.45 |
|  | Erysipelotrichaceae UCG003 | 10.76 | 13 | 0.63 |
|  | Oxalobacter | 5.10 | 10 | 0.88 |
| **Acute tubulo-interstitial nephritis** | Actinomyces | 1.46 | 6 | 0.96 |
|  | Gordonibacter | 9.75 | 9 | 0.37 |
|  | Marvinbryantia | 9.12 | 9 | 0.43 |
|  | Odoribacter | 6.07 | 6 | 0.41 |
| **Chronic tubulo-interstitial nephritis** | Coprococcus3 | 8.47 | 8 | 0.39 |
|  | Dorea | 4.41 | 9 | 0.88 |
|  | Erysipelotrichaceae UCG003 | 8.63 | 13 | 0.80 |
|  | Eubacterium (brachy group) | 8.00 | 9 | 0.53 |
|  | Intestinimonas | 10.21 | 15 | 0.81 |
|  | Victivallis | 7.81 | 9 | 0.55 |
| **Chronic kidney disease** | Butyrivibrio | 15.29 | 14 | 0.36 |
|  | Coprococcus3 | 7.84 | 8 | 0.45 |
|  | Erysipelotrichaceae UCG003 | 10.69 | 13 | 0.64 |
|  | Lachnospira | 3.34 | 4 | 0.50 |
|  | Oxalobacter | 8.12 | 10 | 0.62 |
|  | Prevotella7 | 10.95 | 10 | 0.36 |
|  | Ruminococcus2 | 13.47 | 13 | 0.41 |

**Supplemental Table 4. Assessment of horizontal pleiotropy in the connection between gut microbiota and kidney disease using MR Egger regression**

| **Kidney disease** | **Bacterial genus(exposure)** | **Egger_intercept** | **SE** | **P-value** |
| --- | --- | --- | --- | --- |
| **Nephrotic syndrome** | Akkermansia | 0.11 | 0.082 | 0.23 |
|  | Bacteroides | 0.17 | 0.16 | 0.34 |
|  | Christensenellaceae (R-7 group) | 0.08 | 0.12 | 0.54 |
|  | Gordonibacter | -0.14 | 0.12 | 0.27 |
|  | Lachnospiraceae (ND3007 group) | 0.08 | 1.10 | 0.96 |
|  | Oxalobacter | 0.16 | 0.13 | 0.26 |
|  | Rikenellaceae RC9 (gut group) | -0.02 | 0.15 | 0.99 |
|  | Ruminiclostridium5 | -0.007 | 0.10 | 0.94 |
|  | Ruminococcaceae (NK4A214 group) | -0.17 | 0.08 | 0.06 |
|  | Ruminococcaceae UCG004 | 0.05 | 0.13 | 0.71 |
|  | Ruminococcaceae UCG005 | 0.08 | 0.07 | 0.30 |
|  | Ruminococcus (gnavus group) | 0.03 | 0.12 | 0.82 |
| **Membranous nephropathy** | Butyricicoccus | -0.05 | 0.15 | 0.77 |
|  | Butyrivibrio | -0.01 | 0.08 | 0.92 |
|  | Catenibacterium | -0.002 | 0.30 | 0.99 |
|  | Oscillibacter | -0.11 | 0.11 | 0.35 |
|  | Ruminiclostridium5 | -0.04 | 0.07 | 0.56 |
|  | Ruminococcaceae UCG003 | 0.01 | 0.05 | 0.79 |
|  | Ruminococcaceae UCG013 | -0.07 | 0.08 | 0.40 |
| **Glomerulonephritis** | Coprococcus3 | 0.05 | 0.05 | 0.32 |
|  | Erysipelotrichaceae UCG003 | 0.01 | 0.02 | 0.78 |
|  | Oxalobacter | 0.07 | 0.04 | 0.15 |
| **Acute tubulo-interstitial nephritis** | Actinomyces | 0.01 | 0.02 | 0.62 |
|  | Gordonibacter | 0.04 | 0.02 | 0.17 |
|  | Marvinbryantia | 0.01 | 0.03 | 0.84 |
|  | Odoribacter | 0.04 | 0.02 | 0.15 |
| **Chronic tubulo-interstitial nephritis** | Coprococcus3 | 0.15 | 0.12 | 0.28 |
|  | Dorea | -0.004 | 0.07 | 0.95 |
|  | Erysipelotrichaceae UCG003 | -0.003 | 0.06 | 0.95 |
|  | Eubacterium (brachy group) | -0.04 | 0.09 | 0.63 |
|  | Intestinimonas | 0.03 | 0.05 | 0.60 |
|  | Victivallis | 0.20 | 0.16 | 0.26 |
| **Chronic kidney disease** | Butyrivibrio | 0.10 | 0.03 | 0.01 |
|  | Coprococcus3 | 0.03 | 0.05 | 0.54 |
|  | Erysipelotrichaceae UCG003 | -0.01 | 0.02 | 0.64 |
|  | Lachnospira | 0.004 | 0.09 | 0.97 |
|  | Oxalobacter | 0.08 | 0.05 | 0.13 |
|  | Prevotella7 | -0.002 | 0.06 | 0.98 |
|  | Ruminococcus2 | 0.008 | 0.02 | 0.72 |

MR = mendelian randomization.

**Supplemental Table 5. Analyzing the link between gut microbiota and** **kidney disease with MR-PRESSO**

| **Kidney disease** | **Bacterial genus (exposure)** | **MR Analysis** | **Causal Estimate** | **SD** | **T-stat** | **P-value** | **RSS_obs_** | **Global test P-value** | **Remove SNP** |
| --- | --- | --- | --- | --- | --- | --- | --- | --- | --- |
| **Nephrotic syndrome** | Akkermansia | MR-PRESSO | -0.57 | 0.26 | -2.22 | 0.05 | 10.15 | 0.7 |  |
|  | Bacteroides | MR-PRESSO | 1 | 0.43 | 2.3 | 0.05 | 13.3 | 0.34 |  |
|  | Christensenellaceae (R-7 group) | MR-PRESSO | -0.86 | 0.45 | -1.92 | 0.09 | 11 | 0.4 |  |
|  | Gordonibacter | MR-PRESSO | -0.43 | 0.16 | -2.69 | 0.02 | 12.83 | 0.57 |  |
|  | Lachnospiraceae (ND3007 group) | MR-PRESSO | Not enough instrumental variables | | | | | | |
|  | Oxalobacter | MR-PRESSO | 0.62 | 0.16 | 3.95 | 0.0023 | 9.57 | 0.72 |  |
|  | Rikenellaceae RC9 (gut group) | MR-PRESSO | 0.26 | 0.14 | 1.81 | 0.0947 | 11.06 | 0.68 |  |
|  | Ruminiclostridium5 | MR-PRESSO | -0.68 | 0.22 | -3.13 | 0.0074 | 0.69 | 0.93 |  |
|  | Ruminococcaceae (NK4A214 group) | MR-PRESSO | -0.55 | 0.26 | -2.07 | 0.0562 | 13.73 | 0.69 |  |
|  | Ruminococcaceae UCG004 | MR-PRESSO | 0.55 | 0.17 | 3.29 | 0.0072 | 4.74 | 0.97 |  |
|  | Ruminococcaceae UCG005 | MR-PRESSO | 0.46 | 0.33 | 1.39 | 0.1828 | 26.15 | 0.13 |  |
|  | Ruminococcus (gnavus group) | MR-PRESSO | -0.55 | 0.22 | -2.56 | 0.03 | 10.17 | 0.63 |  |
| **Membranous nephropathy** | Butyricicoccus | MR-PRESSO | 0.24 | 0.55 | 0.43 | 0.68 | 17.29 | 0.053 |  |
|  | Butyrivibrio | MR-PRESSO | 0.2 | 0.1 | 2.04 | 0.07 | 10.76 | 0.644 |  |
|  | Catenibacterium | MR-PRESSO | 0.4 | 0.09 | 4.38 | 0.02 | 1.3 | 0.87 |  |
|  | Oscillibacter | MR-PRESSO | -0.55 | 0.26 | -2.13 | 0.06 | 26.32 | 0.02 | 1 |
|  |  | Outlier-corrected MR-PRESSO | -0.37 | 0.22 | -1.72 | 0.12 | -0.37 |  |  |
|  | Ruminiclostridium5 | MR-PRESSO | 0.33 | 0.21 | 1.59 | 0.14 | 12.84 | 0.62 |  |
|  | Ruminococcaceae UCG003 | MR-PRESSO | 0.51 | 0.19 | 2.76 | 0.02 | 9.99 | 0.7 |  |
|  | Ruminococcaceae UCG013 | MR-PRESSO | -0.4 | 0.26 | -1.54 | 0.16 | 13.86 | 0.35 |  |
| **Glomerulonephritis** | Coprococcus3 | MR-PRESSO | 0.28 | 0.13 | 2.15 | 0.06 | 9.9 | 0.59 |  |
|  | Erysipelotrichaceae UCG003 | MR-PRESSO | -0.29 | 0.08 | -3.54 | 0 | 12.61 | 0.76 |  |
|  | Oxalobacter | MR-PRESSO | 0.15 | 0.06 | 2.56 | 0.03 | 12.13 | 0.54 |  |
| **Acute tubulo-interstitial nephritis** | Actinomyces | MR-PRESSO | -0.16 | 0.03 | -5.07 | 0 | 2.37 | 0.97 |  |
|  | Gordonibacter | MR-PRESSO | -0.07 | 0.04 | -1.92 | 0.08 | 17.35 | 0.29 |  |
|  | Marvinbryantia | MR-PRESSO | 0.28 | 0.07 | 4.06 | 0 | 10.95 | 0.58 |  |
|  | Odoribacter | MR-PRESSO | 0.15 | 0.09 | 1.77 | 0.11 | 11.03 | 0.4 |  |
| **Chronic tubulo-interstitial nephritis** | Coprococcus3 | MR-PRESSO | -0.41 | 0.16 | -2.48 | 0.03 | 11.23 | 0.5 |  |
|  | Dorea | MR-PRESSO | -0.71 | 0.32 | -2.26 | 0.05 | 11.61 | 0.58 |  |
|  | Erysipelotrichaceae UCG003 | MR-PRESSO | -0.5 | 0.22 | -2.33 | 0.03 | 12.66 | 0.74 |  |
|  | Eubacterium (brachy group) | MR-PRESSO | 1.12 | 0.35 | 3.23 | 0.01 | 10.19 | 0.54 |  |
|  | Intestinimonas | MR-PRESSO | 0.29 | 0.19 | 1.5 | 0.15 | 16.16 | 0.72 |  |
|  | Victivallis | MR-PRESSO | 0.23 | 0.15 | 1.52 | 0.16 | 14.16 | 0.40 |  |
| **Chronic kidney disease** | Butyrivibrio | MR-PRESSO | 0.11 | 0.05 | 2.18 | 0.05 | 18.58 | 0.38 |  |
|  | Coprococcus3 | MR-PRESSO | 0.26 | 0.14 | 1.85 | 0.1 | 10.42 | 0.49 |  |
|  | Erysipelotrichaceae UCG003 | MR-PRESSO | -0.31 | 0.08 | -3.5 | 0.003 | 12.98 | 0.77 |  |
|  | Lachnospira | MR-PRESSO | -0.41 | 0.16 | -2.6 | 0.05 | 4.8 | 0.68 |  |
|  | Oxalobacter | MR-PRESSO | 0.12 | 0.08 | 1.62 | 0.13 | 16.83 | 0.25 |  |
|  | Prevotella7 | MR-PRESSO | 0.15 | 0.06 | 2.41 | 0.03 | 13.22 | 0.47 |  |
|  | Ruminococcus2 | MR-PRESSO | 0.21 | 0.1 | 2.19 | 0.05 | 16.06 | 0.5 |  |

**Supplemental Table 6. MR estimates for the association between gut microbiota and membranous nephropathy**

| **Bacterial genus(exposure)** | **No. of SNP** | **MR method** | **OR** | **95% CI** | **P-value** |
| --- | --- | --- | --- | --- | --- |
| **Butyricicoccus** | 5 | MR Egger | 4.64 | (0.04, 553.24) | 0.57 |
|  |  | Weighted median | 2.42 | (0.94, 6.28) | 0.08 |
|  |  | IVW | 2.16 | (1.01, 4.62) | 0.048 |
|  |  | Simple mode | 2.67 | (0.77, 9.33) | 0.18 |
|  |  | Weighted mode | 2.65 | (0.76, 9.21) | 0.21 |
| **Butyrivibrio** | 11 | MR Egger | 1.34 | (0.39, 4.60) | 0.66 |
|  |  | Weighted median | 1.23 | (0.91, 1.68) | 0.18 |
|  |  | IVW | 1.25 | (1.00, 1.57) | 0.048 |
|  |  | Simple mode | 1.37 | (0.85, 2.20) | 0.27 |
|  |  | Weighted mode | 1.33 | (0.81, 2.18) | 0.27 |
| **Catenibacterium** | 4 | MR Egger | 1.52 | (0.02, 139.90) | 0.87 |
|  |  | Weighted median | 1.44 | (0.94, 2.21) | 0.09 |
|  |  | IVW | 1.49 | (1.04, 2.13) | 0.03 |
|  |  | Simple mode | 1.36 | (0.77, 2.39) | 0.39 |
|  |  | Weighted mode | 1.38 | (0.80, 2.39) | 0.33 |
| **Oscillibacter** | 9 | MR Egger | 1.77 | (0.15, 20.41) | 0.66 |
|  |  | Weighted median | 0.51 | (0.29, 0.88) | 0.02 |
|  |  | IVW | 0.53 | (0.29, 0.96) | 0.04 |
|  |  | Simple mode | 0.49 | (0.17, 1.41) | 0.24 |
|  |  | Weighted mode | 0.52 | (0.20, 1.40) | 0.26 |
| **Ruminiclostridium5** | 11 | MR Egger | 3.23 | (0.41, 25.30) | 0.29 |
|  |  | Weighted median | 1.58 | (0.81, 3.09) | 0.18 |
|  |  | IVW | 1.74 | (1.05, 2.86) | 0.03 |
|  |  | Simple mode | 1.55 | (0.55, 4.37) | 0.46 |
|  |  | Weighted mode | 1.57 | (0.57, 4.33) | 0.38 |
| **Ruminococcaceae UCG003** | 10 | MR Egger | 1.46 | (0.35, 6.12) | 0.62 |
|  |  | Weighted median | 1.62 | (0.89, 2.96) | 0.12 |
|  |  | IVW | 1.78 | (1.14, 2.76) | 0.01 |
|  |  | Simple mode | 1.37 | (0.53, 3.54) | 0.53 |
|  |  | Weighted mode | 1.43 | (0.57, 3.58) | 0.44 |
| **Ruminococcaceae UCG013** | 8 | MR Egger | 1.56 | (0.16, 15.56) | 0.71 |
|  |  | Weighted median | 0.64 | (0.32, 1.30) | 0.22 |
|  |  | IVW | 0.57 | (0.328, 0.996) | 0.049 |
|  |  | Simple mode | 0.61 | (0.21, 1.80) | 0.40 |
|  |  | Weighted mode | 0.64 | (0.22, 1.90) | 0.42 |

CI = confidence interval; IVW = inverse-variance weighting; MR = mendelian randomization; OR = odds ratio; SNPs = single nucleotide polymorphisms.

**Supplemental Table 7. MR estimates for the association between gut microbiota and glomerulonephritis**

| **Bacterial genus(exposure)** | **No. of SNP** | **MR method** | **OR** | **95% CI** | **P-value** |
| --- | --- | --- | --- | --- | --- |
| **Coprococcus3** | 9 | MR Egger | 0.59 | (0.13, 2.81) | 0.53 |
|  |  | Weighted median | 1.37 | (0.94, 1.98) | 0.13 |
|  |  | IVW | 1.36 | (1.03, 1.81) | 0.03 |
|  |  | Simple mode | 1.43 | (0.83, 2.46) | 0.20 |
|  |  | Weighted mode | 1.42 | (0.85, 2.37) | 0.22 |
| **Erysipelotrichaceae UCG003** | 14 | MR Egger | 0.70 | (0.41, 1.18) | 0.20 |
|  |  | Weighted median | 0.79 | (0.59, 1.05) | 0.10 |
|  |  | IVW | 0.75 | (0.61, 0.91) | 0.004 |
|  |  | Simple mode | 0.77 | (0.48, 1.24) | 0.31 |
|  |  | Weighted mode | 0.80 | (0.50, 1.26) | 0.34 |
| **Oxalobacter** | 11 | MR Egger | 0.76 | (0.42, 1.38) | 0.39 |
|  |  | Weighted median | 1.16 | (0.97, 1.37) | 0.09 |
|  |  | IVW | 1.21 | (1.07, 1.37) | 0.003 |
|  |  | Simple mode | 1.09 | (0.82, 1.45) | 0.55 |
|  |  | Weighted mode | 1.08 | (0.82, 1.42) | 0.60 |

CI = confidence interval; IVW = inverse-variance weighting; MR = mendelian randomization; OR = odds ratio; SNPs = single nucleotide polymorphisms.

**Supplemental Table 8. MR estimates for the association between gut microbiota and acute tubulo-interstitial nephritis**

| **Bacterial genus(exposure)** | **No. of SNP** | **MR method** | **OR** | **95% CI** | **P-value** |
| --- | --- | --- | --- | --- | --- |
| **Actinomyces** | 7 | MR Egger | 0.78 | (0.57, 1.07) | 0.18 |
|  |  | Weighted median | 0.86 | (0.74, 1.01) | 0.09 |
|  |  | IVW | 0.84 | (0.74, 0.94) | 0.01 |
|  |  | Simple mode | 0.89 | (0.71, 1.11) | 0.34 |
|  |  | Weighted mode | 0.88 | (0.70, 1.11) | 0.30 |
| **Gordonibacter** | 10 | MR Egger | 0.70 | (0.51, 0.97) | 0.07 |
|  |  | Weighted median | 0.91 | (0.81, 1.02) | 0.10 |
|  |  | IVW | 0.90 | (0.82, 0.97) | 0.01 |
|  |  | Simple mode | 0.88 | (0.74, 1.04) | 0.17 |
|  |  | Weighted mode | 0.88 | (0.73, 1.05) | 0.16 |
| **Marvinbryantia** | 10 | MR Egger | 1.23 | (0.67, 2.29) | 0.52 |
|  |  | Weighted median | 1.32 | (1.07, 1.62) | 0.01 |
|  |  | IVW | 1.31 | (1.13, 1.52) | 0.0003 |
|  |  | Simple mode | 1.35 | (1.00, 1.82) | 0.09 |
|  |  | Weighted mode | 1.35 | (1.00, 1.83) | 0.07 |
| **Odoribacter** | 7 | MR Egger | 0.78 | (0.44, 1.38) | 0.43 |
|  |  | Weighted median | 1.32 | (1.04, 1.68) | 0.02 |
|  |  | IVW | 1.23 | (1.03, 1.48) | 0.02 |
|  |  | Simple mode | 1.32 | (0.93, 1.89) | 0.18 |
|  |  | Weighted mode | 1.32 | (0.94, 1.86) | 0.16 |

CI = confidence interval; IVW = inverse-variance weighting; MR = mendelian randomization; OR = odds ratio; SNPs = single nucleotide polymorphisms.

**Supplemental Table 9. MR estimates for the association between gut microbiota and chronic tubulo-interstitial nephritis**

| **Bacterial genus(exposure)** | **No. of SNP** | **MR method** | **OR** | **95% CI** | **P-value** |
| --- | --- | --- | --- | --- | --- |
| **Coprococcus3** | 9 | MR Egger | 0.26 | (0.00, 16.17) | 0.54 |
|  |  | Weighted median | 3.41 | (1.20, 9.67) | 0.02 |
|  |  | IVW | 2.90 | (1.35, 6.22) | 0.01 |
|  |  | Simple mode | 5.22 | (1.02, 26.60) | 0.07 |
|  |  | Weighted mode | 4.80 | (1.14, 20.14) | 0.07 |
| **Dorea** | 10 | MR Egger | 0.49 | (0.07, 3.57) | 0.50 |
|  |  | Weighted median | 0.54 | (0.21, 1.42) | 0.23 |
|  |  | IVW | 0.46 | (0.22, 0.93) | 0.03 |
|  |  | Simple mode | 0.68 | (0.15, 3.06) | 0.63 |
|  |  | Weighted mode | 0.66 | (0.17, 2.50) | 0.58 |
| **Erysipelotrichaceae UCG003** | 14 | MR Egger | 0.55 | (0.14, 2.17) | 0.41 |
|  |  | Weighted median | 0.57 | (0.28, 1.15) | 0.11 |
|  |  | IVW | 0.53 | (0.31, 0.89) | 0.02 |
|  |  | Simple mode | 0.73 | (0.19, 2.79) | 0.66 |
|  |  | Weighted mode | 0.68 | (0.22, 2.06) | 0.50 |
| **Eubacterium (brachy group)** | 10 | MR Egger | 0.99 | (0.25, 4.04) | 0.99 |
|  |  | Weighted median | 0.72 | (0.44, 1.19) | 0.19 |
|  |  | IVW | 0.70 | (0.50, 1.00) | 0.048 |
|  |  | Simple mode | 0.51 | (0.23, 1.15) | 0.13 |
|  |  | Weighted mode | 0.53 | (0.24, 1.18) | 0.17 |
| **Intestinimonas** | 17 | MR Egger | 1.15 | (0.33, 3.96) | 0.83 |
|  |  | Weighted median | 1.27 | (0.70, 2.31) | 0.44 |
|  |  | IVW | 1.58 | (1.01, 2.47) | 0.047 |
|  |  | Simple mode | 1.14 | (0.41, 3.19) | 0.82 |
|  |  | Weighted mode | 1.16 | (0.46, 2.95) | 0.76 |
| **Victivallis** | 10 | MR Egger | 0.32 | (0.03, 3.77) | 0.39 |
|  |  | Weighted median | 1.57 | (1.01, 2.42) | 0.03 |
|  |  | IVW | 1.44 | (1.04, 1.99) | 0.03 |
|  |  | Simple mode | 1.67 | (0.87, 3.21) | 0.18 |
|  |  | Weighted mode | 1.62 | (0.84, 3.09) | 0.22 |

CI = confidence interval; IVW = inverse-variance weighting; MR = mendelian randomization; OR = odds ratio; SNPs = single nucleotide polymorphisms.

**Supplemental Table 10. MR estimates for the association between gut microbiota and chronic kidney disease**

| **Bacterial genus(exposure)** | **No. of SNP** | **MR method** | **OR** | **95% CI** | **P-value** |
| --- | --- | --- | --- | --- | --- |
| **Butyrivibrio** | 15 | MR Egger | 0.57 | (0.36, 0.91) | 0.03 |
|  |  | Weighted median | 1.14 | (0.98, 1.32) | 0.08 |
|  |  | IVW | 1.11 | (1.00, 1.24) | 0.049 |
|  |  | Simple mode | 1.16 | (0.90, 1.50) | 0.28 |
|  |  | Weighted mode | 1.16 | (0.89, 1.51) | 0.29 |
| **Coprococcus3** | 9 | MR Egger | 0.78 | (0.14, 4.35) | 0.78 |
|  |  | Weighted median | 1.24 | (0.80, 1.91) | 0.31 |
|  |  | IVW | 1.36 | (1.00, 1.85) | 0.047 |
|  |  | Simple mode | 1.09 | (0.57, 2.06) | 0.80 |
|  |  | Weighted mode | 1.12 | (0.61, 2.06) | 0.72 |
| **Erysipelotrichaceae UCG003** | 14 | MR Egger | 0.82 | (0.47, 1.45) | 0.52 |
|  |  | Weighted median | 0.69 | (0.51, 0.93) | 0.01 |
|  |  | IVW | 0.72 | (0.58, 0.90) | 0.003 |
|  |  | Simple mode | 1.01 | (0.59, 1.73) | 0.98 |
|  |  | Weighted mode | 1.01 | (0.58, 1.76) | 0.97 |
| **Lachnospira** | 5 | MR Egger | 0.59 | (0.03, 10.75) | 0.75 |
|  |  | Weighted median | 0.68 | (0.40, 1.16) | 0.14 |
|  |  | IVW | 0.64 | (0.42, 0.96) | 0.03 |
|  |  | Simple mode | 0.66 | (0.31, 1.40) | 0.30 |
|  |  | Weighted mode | 0.69 | (0.34, 1.39) | 0.37 |
| **Oxalobacter** | 11 | MR Egger | 0.70 | (0.37, 1.32) | 0.30 |
|  |  | Weighted median | 1.14 | (0.95, 1.38) | 0.17 |
|  |  | IVW | 1.19 | (1.04, 1.36) | 0.01 |
|  |  | Simple mode | 1.05 | (0.76, 1.45) | 0.80 |
|  |  | Weighted mode | 1.04 | (0.76, 1.44) | 0.80 |
| **Prevotella7** | 11 | MR Egger | 1.18 | (0.52, 2.69) | 0.71 |
|  |  | Weighted median | 1.23 | (1.03, 1.48) | 0.02 |
|  |  | IVW | 1.16 | (1.02, 1.33) | 0.03 |
|  |  | Simple mode | 1.27 | (0.92, 1.75) | 0.20 |
|  |  | Weighted mode | 1.27 | (0.89, 1.82) | 0.21 |
| **Ruminococcus2** | 14 | MR Egger | 1.14 | (0.69, 1.88) | 0.61 |
|  |  | Weighted median | 1.35 | (1.01, 1.82) | 0.04 |
|  |  | IVW | 1.24 | (1.01, 1.53) | 0.04 |
|  |  | Simple mode | 1.61 | (0.94, 2.74) | 0.10 |
|  |  | Weighted mode | 1.62 | (1.03, 2.54) | 0.06 |

CI = confidence interval; IVW = inverse-variance weighting; MR = mendelian randomization; OR = odds ratio; SNPs = single nucleotide polymorphisms.

**Supplemental Table 11. Reverse MR estimates for the association between gut microbiota and kidney disease**

| **Kidney disease(Exposure)** | **Bacterial genus(Outcome)** | **No. of SNP** | **MR method** | **OR** | **95% CI** | **P-value** |
| --- | --- | --- | --- | --- | --- | --- |
| **Nephrotic syndrome** | Akkermansia | 6 | IVW | 0.98 | (0.95, 1.02) | 0.30 |
|  | Bacteroides | 6 | IVW | 1.00 | (0.98, 1.03) | 0.74 |
|  | Christensenellaceae (R-7 group) | 6 | IVW | 1.00 | (0.97, 1.03) | 0.95 |
|  | Gordonibacter | 6 | IVW | 0.95 | (0.89, 1.02) | 0.16 |
|  | Lachnospiraceae (ND3007 group) | 6 | IVW | 1.01 | (0.98, 1.05) | 0.48 |
|  | Oxalobacter | 6 | IVW | 1.01 | (0.96, 1.07) | 0.69 |
|  | Rikenellaceae RC9 (gut group) | 0 | IVW | - | - | - |
|  | Ruminiclostridium5 | 0 | IVW | - | - | - |
|  | Ruminococcaceae (NK4A214 group) | 6 | IVW | 1.02 | (0.99, 1.05) | 0.18 |
|  | Ruminococcaceae UCG004 | 6 | IVW | 0.98 | (0.94, 1.02) | 0.31 |
|  | Ruminococcaceae UCG005 | 6 | IVW | 0.98 | (0.94, 1.02) | 0.31 |
|  | Ruminococcus (gnavus group) | 6 | IVW | 1.01 | (0.96, 1.06) | 0.84 |
| **Membranous nephropathy** | Butyricicoccus | 4 | IVW | 1.00 | (0.97,1.02) | 0.79 |
|  | Butyrivibrio | 3 | IVW | 1.01 | (0.89,1.15) | 0.89 |
|  | Catenibacterium | 3 | IVW | 0.97 | (0.92,1.03) | 0.34 |
|  | Oscillibacter | 4 | IVW | 1.02 | (1.00,1.05) | 0.07 |
|  | Ruminiclostridium5 | 4 | IVW | 1.00 | (0.98,1.02) | 0.71 |
|  | Ruminococcaceae UCG003 | 4 | IVW | 0.99 | (0.97,1.00) | 0.14 |
|  | Ruminococcaceae UCG013 | 4 | IVW | 0.99 | (0.97,1.01) | 0.22 |
| **Glomerulonephritis** | Coprococcus3 | 4 | IVW | 1.01 | (0.99, 1.03) | 0.38 |
|  | Erysipelotrichaceae UCG003 | 0 | IVW | - | - | - |
|  | Oxalobacter | 4 | IVW | 0.99 | (0.95, 1.02) | 0.46 |
| **Acute tubulo-interstitial nephritis** | Actinomyces | 2 | IVW | 0.92 | (0.72,1.18) | 0.51 |
|  | Gordonibacter | 0 | IVW | - | - | - |
|  | Marvinbryantia | 2 | IVW | 0.89 | (0.73,1.07) | 0.22 |
|  | Odoribacter | 2 | IVW | 1.01 | (0.84,1.20) | 0.95 |
| **Chronic tubulo-interstitial nephritis** | Coprococcus3 | 2 | IVW | 1.04 | (0.94,1.14) | 0.50 |
|  | Dorea | 2 | IVW | 1.11 | (0.96,1.28) | 0.18 |
|  | Erysipelotrichaceae UCG003 | 0 | IVW | - | - | - |
|  | Eubacterium (brachy group) | 2 | IVW | 1.16 | (0.95,1.42) | 0.13 |
|  | Intestinimonas | 2 | IVW | 0.93 | (0.83,1.05) | 0.24 |
|  | Victivallis | - | IVW | - | - | - |
| **Chronic kidney disease** | Butyrivibrio | 14 | IVW | 0.99 | (0.89, 1.11) | 0.90 |
|  | Coprococcus3 | 18 | IVW | 0.99 | (0.95, 1.04) | 0.74 |
|  | Erysipelotrichaceae UCG003 | 0 | IVW | - | - | - |
|  | Lachnospira | 0 | IVW | - | - | - |
|  | Oxalobacter | 17 | IVW | 0.99 | (0.90, 1.09) | 0.83 |
|  | Prevotella7 | 14 | IVW | 1.03 | (0.90, 1.18) | 0.68 |
|  | Ruminococcus2 | 18 | IVW | 1.01 | (0.96, 1.06) | 0.69 |

CI = confidence interval; IVW = inverse-variance weighting; MR = mendelian randomization; OR = odds ratio; SNPs = single nucleotide polymorphisms.
